# Review of the effects of two major plastic compounds in arthropods: a call for increased interdisciplinarity and further studies at the population level

**DOI:** 10.1007/s11356-025-36773-x

**Published:** 2025-09-24

**Authors:** Amandine Aviles, David Siaussat

**Affiliations:** 1 Département d’Écologie Sensorielle, Sorbonne Université – Institut d’Écologie et des Sciences de l’Environnement de Paris (iEES Paris), Campus Pierre Et Marie Curie (UPMC), 75252 Paris, Cedex 05 France; 2 Maladies infectieuses et Vecteurs: Ecologie, Génétique, Evolution et Contrôle (MiVEGEC), CNRS - IRD - University of Montpellier - INRAE, Montpellier, France; 3 Department of Biology, University of Southern Denmark, 5230 Odense M, Denmark

**Keywords:** Arthropods, Bis(2-ethylhexyl) phthalate, Bisphenol A, Plastics, Biological functions, Behaviour

## Abstract

Due to the massive use of plastics worldwide, plastic chemicals such as bis(2-ethylhexyl) phthalate (DEHP) and bisphenol A (BPA) are ubiquitous in the environment. Concerns about human health promoted studies on the effects of those two chemicals on vertebrates, where they have been found to act as endocrine-disrupting chemicals (EDCs), thus affecting important biological functions under hormonal regulation (e.g. development and reproduction). While arthropods represent most of animal biodiversity and play crucial roles in aquatic and terrestrial environments, few studies have investigated DEHP and BPA effects on those organisms as compared to vertebrates. In this context, the present article aims at reviewing the known effects of DEHP and BPA on arthropods. DEHP and BPA affect various biological functions in arthropods, and could act at low doses and possibly induce long-term effects for organisms, but the mechanisms of actions of those chemicals in arthropods are still unknown. We discuss research gaps on this issue and ecotoxicology in arthropods in general: (1) the notion of DEHP and BPA as endocrine disruptors in arthropods; (2) the usefulness of behavioural studies in arthropods’ ecotoxicology; (3) the need to better understand epigenetic mechanisms in arthropods and how chemicals could interfere with them; (4) the inclusion of the effects on arthropod's microbiota in ecotoxicological studies; and (5) the need to increase the use of integrative approaches, omics, modelling and adverse outcome pathways to better understand chemicals’ mechanisms of actions in arthropods and the resulted adverse outcomes at population level.

**Graphical Abstract:**

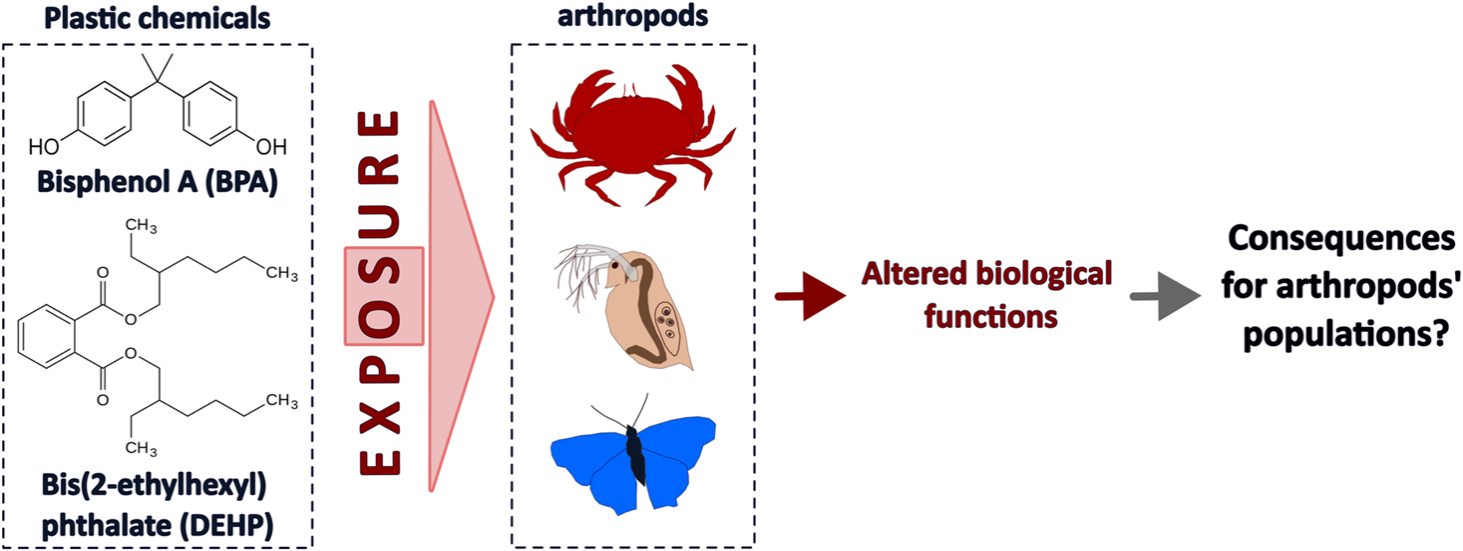

**Supplementary Information:**

The online version contains supplementary material available at 10.1007/s11356-025-36773-x.

## Introduction

### Massive production and consumption of plastics for decades

Recent data show that, while plastic production is stable in Europe (58.1 Mt In 2018, 53.9 Mt In 2020 and 57.2 In 2021), it is still increasing worldwide (from 365.5 to 390.7 Mt between 2018 and 2021) (Plastic Europe 2022). Europe still represents 15% of worldwide plastic production In 2021 (Plastic Europe 2022). In terms of economic added value, plastic Industry is the 8th most important industry in Europe, similar to electrical equipment, and close to the pharmaceutical industry. In terms of usages, packaging and building and construction represent the Main end-use Markets of plastic, representing respectively 39.1% and 21.3% of the Market In 2021 (Plastic Europe 2022). In view of the still massive use and production of plastic worldwide, the question of the environmental pollution of plastics, and their added chemicals, remains a critical issue.

### DEHP and BPA are ubiquitous in the environment

Bis(2-ethylhexyl) phthalate (DEHP) and bisphenol A (BPA) remain two of the most used plastic compounds, and are therefore ubiquitous in the environment.

Phthalates are broadly used plasticisers, whose production was estimated to 6 million tons per year In 2006. They are used in polyvinyl chloride (PVC) products (e.g. building materials, medical equipment, toys), and other everyday-life products like perfumes, cosmetics, adhesives and paints (EU 2008). DEHP is one of the most used phthalate (Gao and Wen 2016), and is consequently present everywhere in the environment, along with its main metabolite monoethylhexyl phthalate (MEHP) (Bergé et al. 2013). DEHP is poorly soluble in water and tends to accumulate in soils and sediments due to its high octanol/water coefficient (between 4.8 and 9.6) (EU 2008) (Table 1 and supplementary data S1). Concentrations in agricultural and urban soils Range from 0.02 to 264 mg/kg, and are on average 10 mg/kg (dw) in sediments (Table 1 and supplementary data S1). DEHP is poorly degraded under anaerobic conditions and its Half-life was estimated to be 2 to 5 weeks in water (Corrales et al. 2015), and over 100 years in sediments (Yan et al. 1995).

**Table 1:** Overview of BPA and DEHP distributions in the environment

	**BPA**	**References**	**DEHP**	**References**
Atmosphere	1–17,400 pg/m^3^	Graziani et al. 2019; Huang et al. 2012; Michalowicz 2014; Wu et al. 2018	0.22 pg/m3–3100 ng/m^3^	Clark et al. 2003; Ebinghaus and Xie 2006; Gao and Wen 2016; Lee et al. 2019; Xie et al. 2005
Drinking water	n.d.–4.21 μg/L	Corrales et al. 2015; Flint et al. 2012; Huang et al. 2012; Kmiecik et al. 2020; Michalowicz 2014; Zhang et al. 2016	n.d.–172 μg/L	Abtahi et al. 2019; Clark et al. 2003; Gani et al. 2017; Horn et al. 2004; Zolfaghari et al. 2014
Freshwater	n.d.–56 μg/L	Chen et al. 2014; Corrales et al. 2015; Flint et al. 2012; Huang et al. 2012; Kang et al. 2007; Li et al. 2015; Lu et al. 2020; Michalowicz 2014; Wu et al. 2018; Xu et al. 2014; Zhang et al. 2016	n.d.–336 μg/L	Abtahi et al. 2019; Clark et al. 2003; Gani et al. 2017; D. W. Gao and Wen 2016; Horn et al. 2004; Magdouli et al. 2013; Peijnenburg and Jaap 2006; Wang et al. 2005; Zolfaghari et al. 2014
Seawater	< 0.04 ng/L–2.47 μg/L	Corrales et al. 2015; Flint et al. 2012; Hermabessiere et al. 2017; Huang et al. 2012; Kang et al. 2007	0.16 ng/L–64.3 μg/L	Ebinghaus and Xie. 2006; Hermabessiere et al. 2017; Xie et al. 2005; Zolfaghari et al. 2014
Sediments	n.d.–13.37 mg/kg	Arditsoglou and Voutsa, 2012; Arukwe et al., 2012; Chen et al. 2014; Corrales et al. 2015; Flint et al. 2012; Hermabessiere et al. 2017; Huang et al. 2012; Jahromi et al. 2020; Kang et al. 2007; Stewart et al. 2014; Wu et al. 2018	n.d.–487 mg/kg	Clark et al. 2003; D. W. Gao and Wen 2016; Horn et al. 2004; Lee et al. 2019; Oehlmann et al. 2008; Sirivithayapakorn and Thuyviang 2010; Stewart et al. 2014; Zolfaghari et al. 2014
Soil	n.d.–1000 μg/kg	Corrales et al. 2015; Flint et al. 2012; Tran et al. 2015a; S. Wang et al. 2018; Zhang et al. 2015	n.d.–63 mg/kg	Clark et al. 2003; D. W. Gao and Wen 2016; Hongjun et al. 2013; Ma et al. 2013; Tran et al. 2015b; Wang et al. 2020; Xu et al. 2008; Zhou et al. 2020
Landfill leachate	< 1.3–17,200 μg/L	Flint et al. 2012; Huang et al. 2012; Michalowicz 2014	9.6–460 μg/L	Horn et al. 2004; Zolfaghari et al. 2014
Sewage sludge	4 μg/kg–32,000 mg/kg	Corrales et al. 2015; Flint et al. 2012; Huang et al. 2012; Michalowicz 2014; Tran et al. 2018	1.8–1340 mg/kg	Magdouli et al. 2013; Zolfaghari et al. 2014; Tran et al. 2018
Plants (field sampling)	< LOQ	Lo Turco et al. 2020	0.23–1.18 μg/kg	Di Bella et al. 2018; Turco et al. 2020
Plants (exposed crops)	0.22–7000 μg/kg (crops exposed from 50 ng/L to 20 mg/L)	Ahammed et al. 2020; Dodgen et al. 2013; *Kanwar *et al*. *2020*;* Lu et al. 2012, 2014; Zhang et al. 2015	0.5–362 mg/kg	Ma et al. 2013; Sablayrolles et al. 2005; Sun et al. 2015

Bisphenol A or BPA is a monomer mostly used in polycarbonate plastics, which are found in many common consumer products Such as bottle for drinks and reusable tableware. BPA is also found In some epoxy resins, used to coat the Inside of cans for food and drinks and water pipes, and in dies for thermal paper. The estimated BPA production and consumption in the UE In 2005/2006 were around 1 million tons (EU 2008). While BPA is mostly used as a plastic monomer and not a plasticiser like DEHP, heat, basic or acid conditions, and repeated washing of consumer products can result in the hydrolysis of the ester bonds between BPA molecules, and therefore the release of those molecules in the environment (Corrales et al. 2015). BPA is poorly present in the atmosphere but is found in Surface water at concentrations up to 56 μg/L, with a mean concentration In tap water of 14 ng/L (supplementary data S1). Due to a high octanol/water partitioning coefficient, BPA is mostly found in soils (up to 1000 μg/kg) and sediments (between 100 and 1000 μg/kg downstream of heavily populated urban areas, WWTPs or industrial discharges) (Table 1).

### DEHP and BPA have been regulated due to their effects on humans

Both DEHP and BPA have recognised adverse effects in humans, including on reproduction and neurodevelopment (Liu et al. 2023; Ma et al. 2019; Vom Saal and Vandenberg 2021; Wen et al. 2022). In Europe, DEHP is considered a cancerogenous, mutagenous and toxic for reproduction (CMR) substance (European Chemical Agency (ECHA), 2012, 2010; European Environment Agency (EEA), 2012), and as an endocrine disruptor (ECHA 2025a). DEHP is listed as a Substance of Very High Concern (SVHC) under the REACH regulation, and is submitted to authorisation in Europe (ECHA, 2025a). DEHP use is banned in toys, cosmetics and personal care products in Europe (European Chemical Agency (ECHA), 2010). DEHP has been classified by the EU as a priority substance with an environmental quality standard (EQS) of 1.3 μg/L in water (EU 2008). While DEHP regulation has Led to a decreased consumption, DEHP still represents 10% of the European plasticiser Market and 37% of the global plasticiser Market In 2015 (ECPI 2018). In the United States, the Environmental Protection Agency (US EPA) has designated DEHP as a High Priority Substance In December 2019, resulting in DEHP being at present subjected to risk evaluation (US EPA 2025). In China, DEHP is prohibited In cosmetics and restricted in plastic toys to up to 0.1% of material composition (Monti et al. 2022). BPA has been classified as a Substance of Very High Concern (European Chemical Agency (ECHA), 2018) In the EU. It has been banned from plastic bottles and packaging containing food for babies and children under 3 years old and, more recently, from food contact materials (ECHA 2025b); European Commission 2025). In the United States, the Food and Drug Administration (FDA) deauthorised the use of BPA for baby bottles, sippy cups and packaging for Infant formulas In 2012 and 2013 (FDA, 2025). In China, the government banned the sale and import of baby bottles containing BPA In 2011, and restricted BPA use In plastics In contact with food In 2011 (Wang et al., 2025).

### Aims of the review

While DEHP and BPA are ubiquitous in the environment, and arthropods have critical roles in aquatic and terrestrial ecosystem (e.g. pollination, organic carbon recycling), few studies have been conducted on the effects of those compounds on arthropods as compared to vertebrates. In this review, we try to summarise current knowledge on the effects of those two plastic compounds on arthropods, in view of helping future research in this area. More particularly, this review is structured around two main parts:

1. Reviewing the literature on the effects of DEHP and BPA on arthropod organisms.
2. Discussing several gaps and areas of investigation regarding the effects of DEHP and BPA in arthropods and ecotoxicology in arthropods in general.

## Short description of the methodology

The review of the literature on the effects of BPA and DEHP on arthropods was performed between May 2017 and December 2023 using Google scholar and Web of Science databases. The search was conducted using a combination of keywords from the two following lists: (i) “plasticizer”, “phthalates”, “Bis(2-ethylhexyl) phthalate”, “DEHP”, “Bisphenol A”, “BPA”, and (ii) “invertebrates”, “arthropods”, “insects”, “crustaceans”, “*Drosophila*”, “*Daphnia*”, “*Chironomus*”. For this review, we decided to focus on articles performing experiments on the whole organism, and investigating chronic endpoints (i.e. articles only focusing on determining a lethal concentration or dose were not selected).

To critically assess the quality of the articles, we used the “Criteria for Reporting and evaluating Ecotoxicity Data” (CRED), described in Moermond et al. (2016). We decided to use this tool as a guidance to assess the quality and not in view of doing a thorough environmental assessment of the effects of DEHP and BPA on the environment. This article does not aim to be of use for regulatory purpose, but only to Summarise current knowledge and gaps regarding the effects of DEHP and BPA on arthropods in order to help guiding further research effort in this area. The CRED criteria were designed for aquatic ecotoxicity data, but they can also be used for terrestrial ecotoxicity data. The CRED method can be used to evaluate both relevance and reliability of an article. However, in the present article, the relevance of the articles was already assessed while reviewing the Literature, and the CRED criteria were only used to assess the reliability of the articles. There are 50 reliability criteria, organised in 6 classes: test setup, test compound, test organism, exposure conditions, statistical design and biological response. The overall reliability of an article can then be categorised as either: reliable without restrictions (R1), reliable with restrictions (R2), not reliable (R3), and not assignable (i.e. important information missing, R4). The resulting Excel sheets comprising the reliability assessment of the articles used in this review are available in supplementary data S2. Because of the number of articles taken into consideration, we did not contact the authors when information was missing and conducted the assessment based on the information available in the article and the supplementary data. Besides, when a specific guideline was used for one or several experiments, we did not check if the criteria of the guideline were respected, but, when possible, indicated the name of the guideline as a comment in the table.

Due to the high diversity in arthropods, in particular regarding their biological organisation, it is difficult to extrapolate the results of an experiment made with one taxon to another one. For instance, “crustaceans” is a paraphyletic group. According to recent molecular phylogenies, some crustaceans like Branchiopods (e.g. Daphnies) are now considered being more closely related to hexapods than decapods (Giribet and Edgecombe 2019). Therefore, to prevent any confusion and avoid inappropriate interpretations, we tried to specify at best on which species the experiments were conducted.

## Plastic chemicals’ effects on arthropods

The literature on the effects of DEHP and BPA in arthropods is presented in supplementary data S2. We summarise the main results in the following.

### DEHP effects in arthropods

**Fig. 1:**
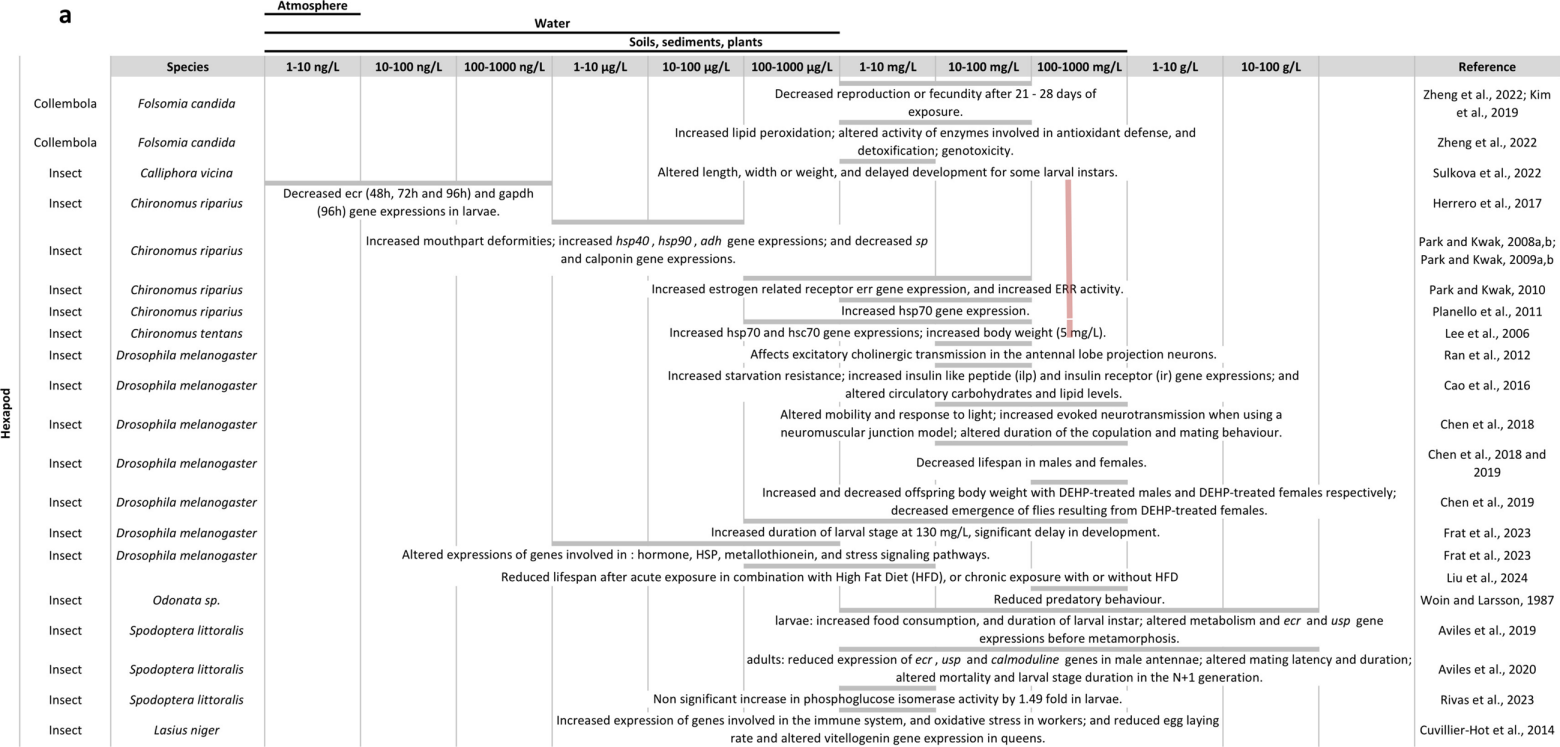

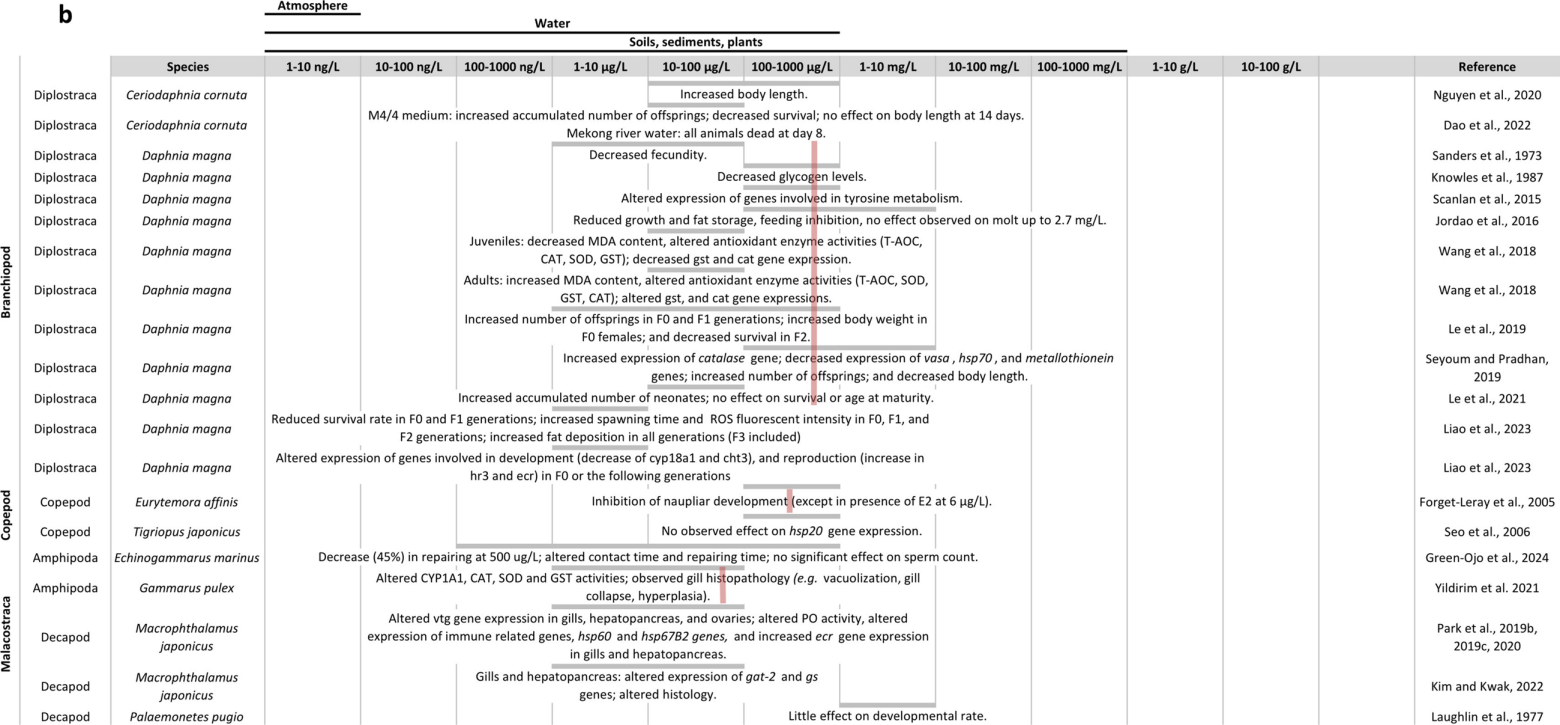
Overview of the literature on DEHP effects in arthropods: **a** in insects, **b** in other arthropods**.** The table is organised with DEHP concentrations in columns and arthropod species and references in rows. At each intersection, the effects found by the considered reference are described. For some species, a pink line is shown representing the mean observed LC50 at 24 h, 48 h or 96 h depending on current knowledge (Supplementary materials S2)

The effects of DEHP exposure on arthropods are summarised in Fig. 1.

#### Toxicity, physiological and biochemical effects

DEHP is present In both terrestrial and aquatic arthropod species. DEHP was detected in 8% of 75 adult honeybees sampled in Spain (Gómez-Ramos et al. 2019). Concentrations measured In ants reached 1 µg/g fresh weight (fw), when including two other phthalates (dibutyl phthalate (DBP), and diisobutyl phthalate (DiBP)) (Lenoir et al. 2012). Internal concentrations of DEHP were 2.9 µg/g fw in dragonfly larvae (Woin and Larsson 1987), between 0.113 and 2.169 µg/g dry weight (dw) in the moth *Spodoptera littoralis* (Aviles et al. 2020), up to 0.212 µg/g fw in prawns (Hu et al. 2016) and 8.9 µg/g dw in crabs (Tiwari et al. 2019).

Measured bioaccumulation factors (BFs) are highly variable, depending on the considered species and stage, and Initial DEHP concentration In the environment. For example, measured BF was 350 in *Chironomus plumosus* exposed to 0.3 µg DEHP/L for 7 days (Mayer and Sanders 1973), but only 1.5 in adult *Chironomus riparius* after exposure during development from 100 to 10,000 mg DEHP/kg of sediment (dry weight) (Brown et al. 1996). Measured BF reached 4108 in *Culex pipiens quinquefasciatus* larvae exposed to 10 ppm (10 mg/L) for 24 h (Metcalf et al. 1973), and 3900 and 3600 in *Gammarus pseudolimneus* exposed to 0.1 µg DEHP/L for 7 and 14 days (Mayer and Sanders 1973). After exposure to 0.3 µg DEHP/L for 7 days, *Daphnia magna*’s BF was 420 (Mayer and Sanders 1973).

Regarding acute toxicity of DEHP, calculated LC50 are also very variable, depending on the species and the stage of exposures (i.e. larvae or adults). DEHP is not very lethal in *Chironomus* sp., where the 24 h LC50 is higher than 100 mg/L (Planelló et al. 2011). Gammarus and Daphnids seem to be more sensitive to DEHP, with 24 h LC50 of 7, 0.83 and 0.48 mg/L respectively in *Gammarus pseudolimneus*, *D. magna* juveniles and *D. magna* adults (Mayer and Sanders 1973; Wang et al. 2018).

DEHP can affect detoxification mechanisms or response to oxidative stress in several species. DEHP induces oxidative stress in ant workers (Cuvillier-Hot et al. 2014), and alters the expression of genes involved in response to oxidative stress in *Daphnia magna* (Seyoum and Pradhan 2019). In *Folsomia candida* raised In soil containing between 4 and 20 mg DEHP/kg, for up to 14 days, DEHP tended to decrease the activity of catalase, GST and AChE and increase lipid peroxidation (Zheng et al. 2022). Increased expression of *heat shock protein* *(hsp)* *genes* following exposure to DEHP has been observed in *Chironomus riparius* (Herrero et al. 2017; Park and Kwak 2008; Planelló et al. 2011), *Chironomus tentans* (Lee et al. 2006), *Drosophila melanogaster* (Frat et al. 2023) and *Macrophthtalamus japonicus* (Park et al. 2020). Exposure to 10 µM DEHP (i.e. around 3.9 mg/L) decreased the expression of *hsp70* and metallothionein genes in *Daphnia magna* (Seyoum and Pradhan 2019), but had no effect on *hsp20* gene expression in *Trigriopus japonicus* (Seo et al. 2006). HSPs are molecular chaperone proteins that are involved in response to stress. In particular, in case of a temperature stress, they protect enzymatic functions and contribute to the folding and conformational regulation of proteins (Feder and Hoffmann 1999 in Agwunobi et al. 2021). In *Gammarus pulex*, DEHP exposure altered the activity of enzymes involved in detoxification (Yildirim et al. 2021). In *D. melanogaster*, chronic exposure to 0.0013 or 0.13 mg DEHP/L can affect the expression of genes involved in metallothionein and stress signaling pathways (Frat et al. 2023).

DEHP can also affect Survival and Lifespan. Exposure to 1 and 2 g DEHP/kg of dry soil for 14 days reduced the survival of *Folsomia candida* (Kim et al. 2019). In *D. melanogaster*, decreased lifespan in Males and females was observed after raising them In a medium containing from 75 to 750 mg DEHP/kg/day (Chen et al. 2019), and after chronic exposure from 1 (around 390 µg/L) to 10 µM (around 3.9 mg/L) DEHP (Liu et al. 2024). Interestingly, Dao et al. (2022) observed that survival of *Ceriodaphnia cornuta* to DEHP exposure for 14 days was also dependent on the type of medium they were raised in (i.e. M4/4 or Mekong river water). In M4/4, medium Survival was 56% after exposure to 100 μg DEHP/L for 14 days, as compared to 88% for controls. In Mekong River water, all Individuals were dead at day 8 (Dao et al. 2022).

Recent articles also showed a general effect on physiology with an observed histopathology of gills in *G. pulex* (Yildirim et al. 2021), and gills and hepatopancreas in *M. japonicus* (Kim and Kwak 2022).

#### Development and metabolism

DEHP interferes with growth and development in different species. In *Chironomus* sp., 1-day exposure to environmental concentrations of DEHP delays development and increases the occurrence of mouthpart deformities (Park and Kwak 2008). In *Spodoptera littoralis*, exposure to a wide range of DEHP concentrations (from 1.1 μg to 39.5 mg/Kg) in food during post-embryonic development increased larval instar duration and affected the development in the next generation (F1) (Aviles et al. 2020, 2019). Raising *Calliphora vicina*, a diptera used in forensic, on pork Liver with 4.5 mg DEHP/kg, resulted in altered length, width or weight, and delayed development for some larval instars (Sulkova et al. 2022). In copepods, several days of exposure to intermediate concentrations (i.e. 109 µg/L) inhibited naupliar development in *Eurytemora affinis* (Forget-Leray et al. 2005). Several days of exposure to DEHP at concentrations from 50 to 500 µg/L decreased body length in *D. magna* (Le et al. 2019; Seyoum and Pradhan 2019), but contradictory effects have been observed on the body length of *Ceriodaphnia cornuta* (Dao et al. 2022; Nguyen et al. 2020). Little effect was observed on *Palaemonetes pugio* development rate at up to 1 ppm (1 mg/L) (Laughlin et al. 1978).

DEHP also affects carbohydrate and lipid metabolism. In *Spodoptera littoralis*, exposure to a wide range of DEHP concentrations (from 1.1 µg to 39.5 mg/kg) in food during development increased food consumption in larvae, without affecting growth, and altered metabolism during the three last days of the last larval instar (Aviles et al. 2019). In this species, DEHP may also affect glycolysis in the last larval instar by increasing the activity of phosphoglucose isomerase (Rivas et al. 2023). In *Drosophila melanogaster*, chronic exposure to DEHP from 0.13 to 130 mg/L induced a significant delay in post-embryonic development (Frat et al. 2023). Raising flies on food containing 200 µM (around 78.12 mg/kg) DEHP altered circulatory carbohydrate and lipid levels, and increased insulin like peptide (ILP) and insulin receptor (IR) gene expressions (Cao et al. 2016).

In *D. magna*, exposure to DEHP alters growth and fat storage, decreases glycogen levels, and inhibits feeding (Jordao et al. 2016; Knowles et al. 1987).

#### Fecundity, immune system and hormonal pathways

DEHP effects on fecundity have been observed. Exposure to 2 ng/µL once a week for 5 weeks reduced the egg-laying rate and altered vitellogenin gene expression in ant queens (Cuvillier-Hot et al. 2014). In *Macrophthalamus japonicus*, exposure from 1 to 30 µg/L for 96 h increased vitellogenin gene expression in the hepatopancreas and ovaries (Park et al. 2019a). Effects of DEHP on *D. magna* fecundity seem less clear but several studies showed that both short-term (24 h) and long-term (3 generations) exposures increased the number of offsprings (Le et al. 2019, 2021; Seyoum and Pradhan 2019). Increased fecundity was also observed in *C. cornuta* exposed to 100 µg/L DEHP in M4/4 medium for 14 days (Dao et al. 2022). DEHP also affected reproduction in *Folsomia candida*, with a 28-day EC50 of 19.72 mg/kg (Zheng et al. 2022).

Exposure to environmental concentrations of DEHP for up to 7 days also affected the expression of genes involved in the immune system in ants (Cuvillier-Hot et al. 2014) and in *M. japonicus* (Park et al. 2019c).

DEHP can also affect the ecdysteroid pathway. In *D. melanogaster*, exposure to DEHP at 0.0013 mg/L during development resulted in a twofold increase of broad-complex gene expression (Frat et al. 2023). DEHP also decreased the expression of the ecdysteroid receptor (*ecr*) gene in *Chironomus riparius* larvae at low (down to 1 ng/L) (Herrero et al. 2017) and high (100 mg/L) concentrations (Planelló et al. 2011). In *Spodoptera littoralis*, DEHP exposure in food during development altered the expression of *ecdysteroid receptor* (*ecr*) and *ultraspiracle* (*usp*) genes during the 3 last days of the last larval instar (at 4.3 mg/g of food) (Aviles et al. 2019), and decreased the expression of *ecr* in adult males’ antennae (from 676 ng/g of food) (Aviles et al. 2020). The ecdysteroid receptor forms with ultraspiracle, a heterodimer that serves as nuclear receptor binding ecdysteroids (Lafont et al. 2021). In *Macrophthalamus japonicus*, *ecr* gene expression was Increased In gills and hepatopancreas after 1-week exposure to environmental concentrations of DEHP (1 to 30 µg/L) (Park et al. 2019a).

Intergenerational effects were observed in *Daphnia magna* after exposure of parental generation from 24 h old Individuals till spawning to 1 µg/L DEHP (Liao et al. 2023). DEHP exposure reduced survival rate in F0 and F1 generations; increased spawning time and ROS fluorescent intensity in F0, F1 and F2 generations; increased fat deposition in all generations; and altered expression of genes involved in development (i.e. decrease of *cytochrome P450 18a1*-*cyp18a1* and *chitinase* 3-*cht3*), and reproduction (i.e. increase in *hormone receptor 3*-*hr3* and *ecr*) (Liao et al. 2023). Cytochrome P450 are proteins that are involved in many biological functions, including detoxification and hormone metabolism. *Cyp18a1* encodes an enzyme involved in the catabolism of the molting hormone. Cht3 encodes a Chitinase, a glycolhydrolase enzyme that that breaks down glycosidic bonds in chitin. Cht3 and hr3 are downstream genes of fushi Tarazu factor1 (ftz-f1), a nuclear receptor regulated by and involved in the ecdysteroid pathway.

#### Nervous system and behaviour

In *Macrophthalamus japonicus*, exposure to 1 to 30 µg/L DEHP altered the expression of gamma-aminobutyric acid Transporter type 2 and glutamine synthetase (both involved in neurotransmission), in the hepatopancreas and gills (Kim and Kwak 2022). In *D. melanogaster,* DEHP at 0.2 and 0.4% (around 0.985 g/L and 3.94 g/L respectively) was shown to alter neural transmission in neuromuscular junctions and, consistently, reduce motor capacities in both sexes (at 0.4% for males, and in a concentration-dependant way for females) (Chen et al. 2018). Besides, DEHP at 0.1 and 0.4% altered the response to light (Chen et al. 2018), and DEHP at 300 μM (around 117 mg/L) affected excitatory cholinergic transmission in the antennal lobe projection neurons in an in vitro setup (Ran et al. 2012), suggesting that, in this species, DEHP could affect both visual and olfactory systems.

Sexual behaviour was also affected in *D. melanogaster*, where copulation duration was respectively increased and decreased in Males and females treated with 0.4% DEHP and mated with control individuals (Chen et al. 2018). In the same study, for exposure to 0.2 and 0.4% DEHP, control females pre-mated with DEHP-treated males displayed an increased re-mating behaviour (Chen et al. 2018). DEHP also affects sexual behaviour in male *Spodoptera littoralis*, In particular after exposure to 676 ng and 19.7 μg DEHP/g of food (Aviles et al. 2020). Consistently, the antennal expressions of ecdysteroid response genes and of calmodulin, an effector partly involved in olfaction (Bahk and Jones 2016), were reduced at the same concentrations, suggesting that, in this species, DEHP could interfere with olfaction via the ecdysteroid pathway (Aviles et al. 2020; Bigot et al. 2012). Mating was also affected as DEHP-treated females mated with control males displayed a delay In Mating and a shortened mating time from 1.1 μg to 4.3 mg DEHP/g of food (Aviles et al. 2020). In *Echinogammarus marinus*, exposure to DEHP from 0.5 to 500 µg/L for 96 h increased repairing time and decreased repairing in a concentration-dependant way (Green-Ojo et al. 2024a).

Other behaviours than sexual behaviours were also affected by DEHP. Thurén and Woin (1991) showed that high concentration of DEHP (500 mg/L) decreased the locomotor activity in *Gammarus pulex*, but this would probably not be caused by endocrine disruption at such high concentration. Green-Ojo et al. (2024b) investigated the effect of DEHP (from 0.5 to 500 μg/L) on the swimming activity and startle response of the marine crustacean *Echinogammarus marinus*, and found no significant effects of DEHP. Exposure to DEHP also resulted in a reduced predatory behaviour in the dragonfly larvae (Woin and Larsson 1987).

#### Conclusions on DEHP effects in arthropods

The available articles on DEHP In arthropods show that DEHP is found In arthropods and it can affect several biological functions. DEHP affects detoxification mechanisms and oxidative stress in arthropods, and can be toxic at different concentrations, depending on the sensitivity of the considered species. When considering 24 h LC50, *D. magna* is more sensitive to DEHP than *Gammarus pseudolimneus*. DEHP was also found to affect survival and lifespan in several species like *D. melanogaster* and *Ceriodaphnia cornuta*, and induce histopathological effects in gills in *G. pulex* and *M. japonicus*. Arhtropods’ growth, post-embryonic development and fecundity were affected by DEHP exposure. This could result from DEHP effects on carbohydrate and lipid metabolism or a possible interaction with hormonal pathways. Indeed, DEHP affected the expression of the *ecr* gene, involved in the ecdysteroid pathway, for a wide range of concentrations: from 1 ng/L to 100 mg/L In water; and from 676 ng/g to 4.3 mg/g in food. Effects of DEHP on the nervous system were observed in *D. melanogaster* and *M. japonicus*. Behaviour was affected by DEHP in several species, with most studies focussing on sexual behaviours. In *Spodoptera littoralis*, DEHP effects on sexual behaviour may be mediated by the ecdysteroid system, given that decrease in *ecr* gene expression and effects on behaviour were observed at similar DEHP concentrations. However, more studies on DEHP mechanisms of action in arthropods are necessary to confirm this hypothesis.

### BPA effects in arthropods

**Fig. 2:**
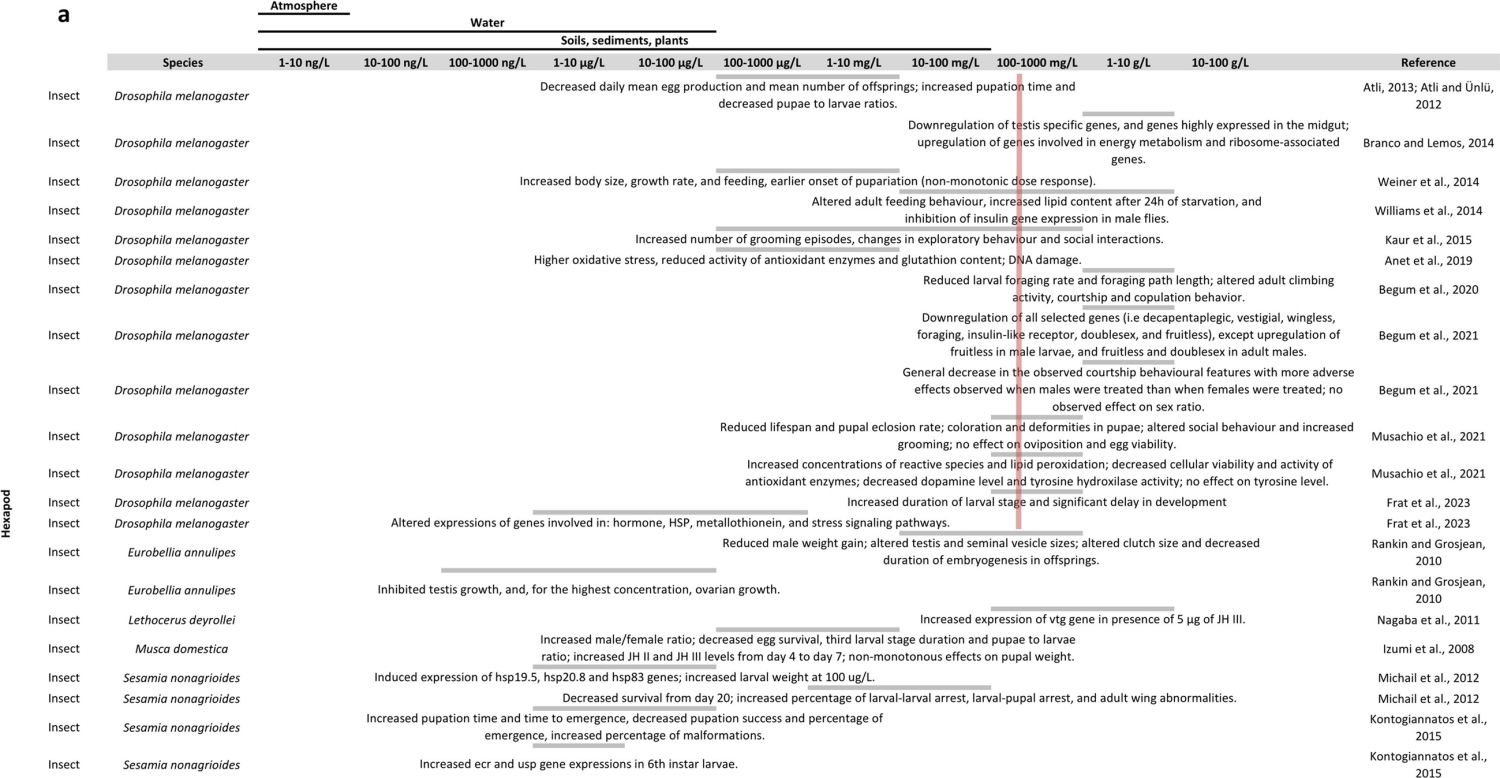

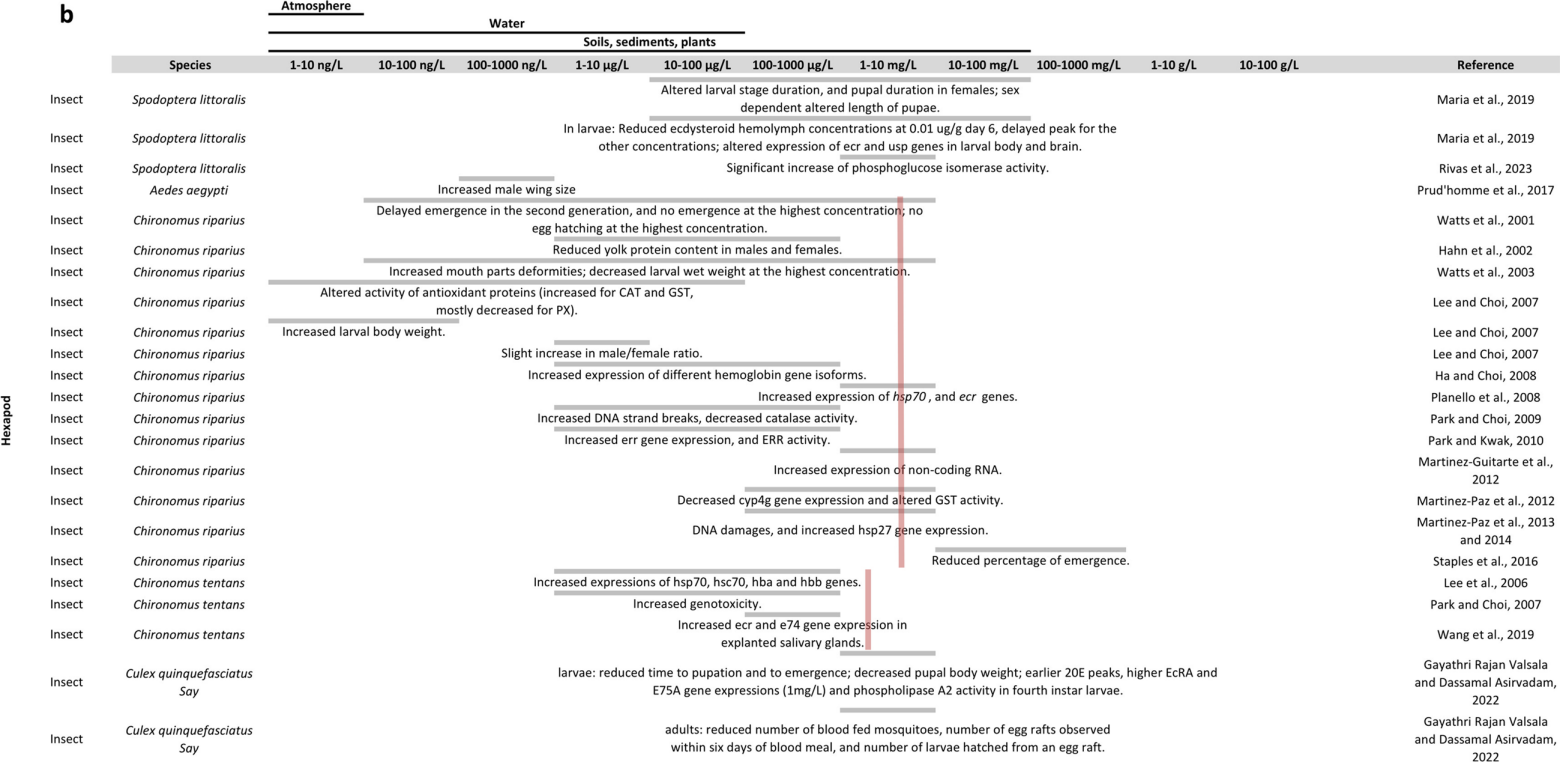

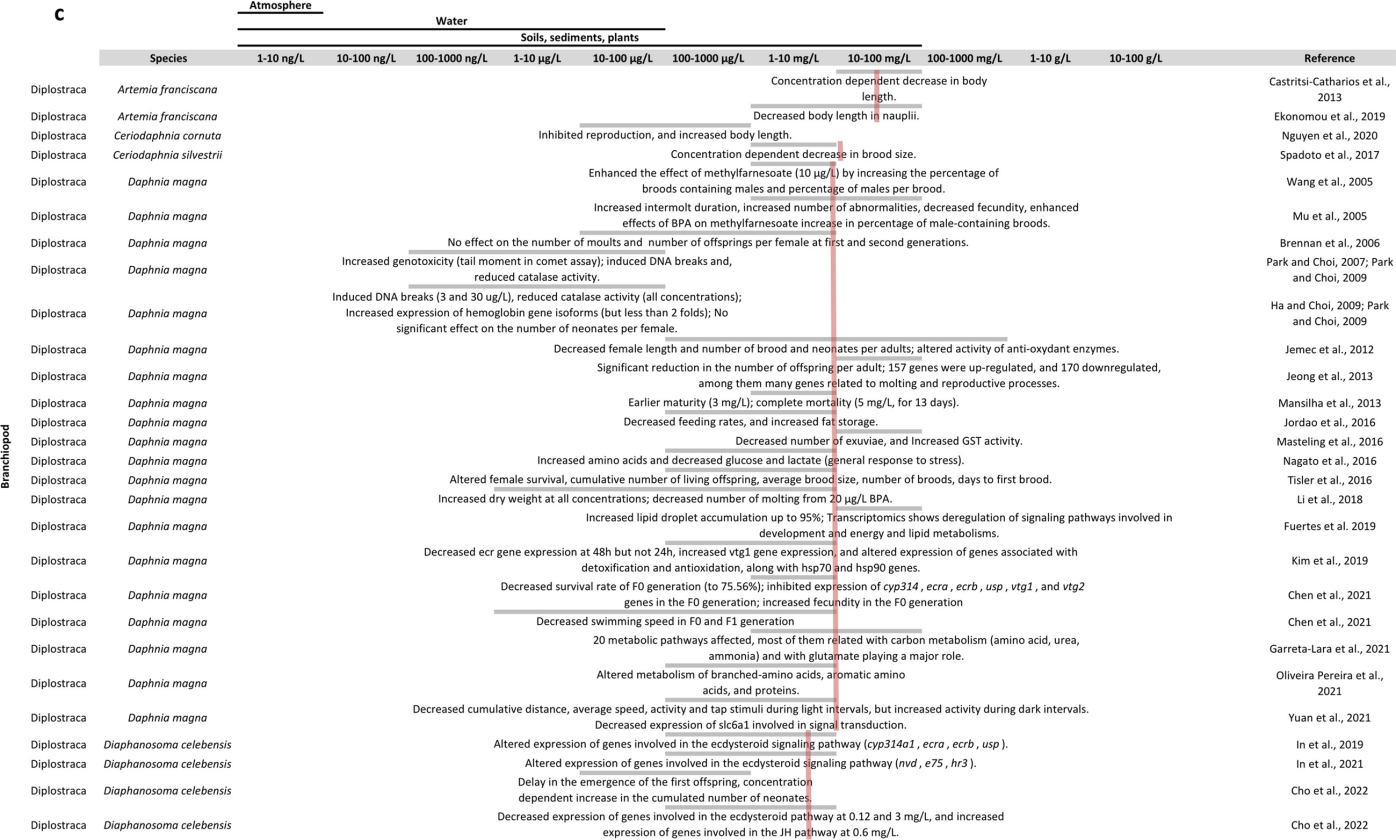

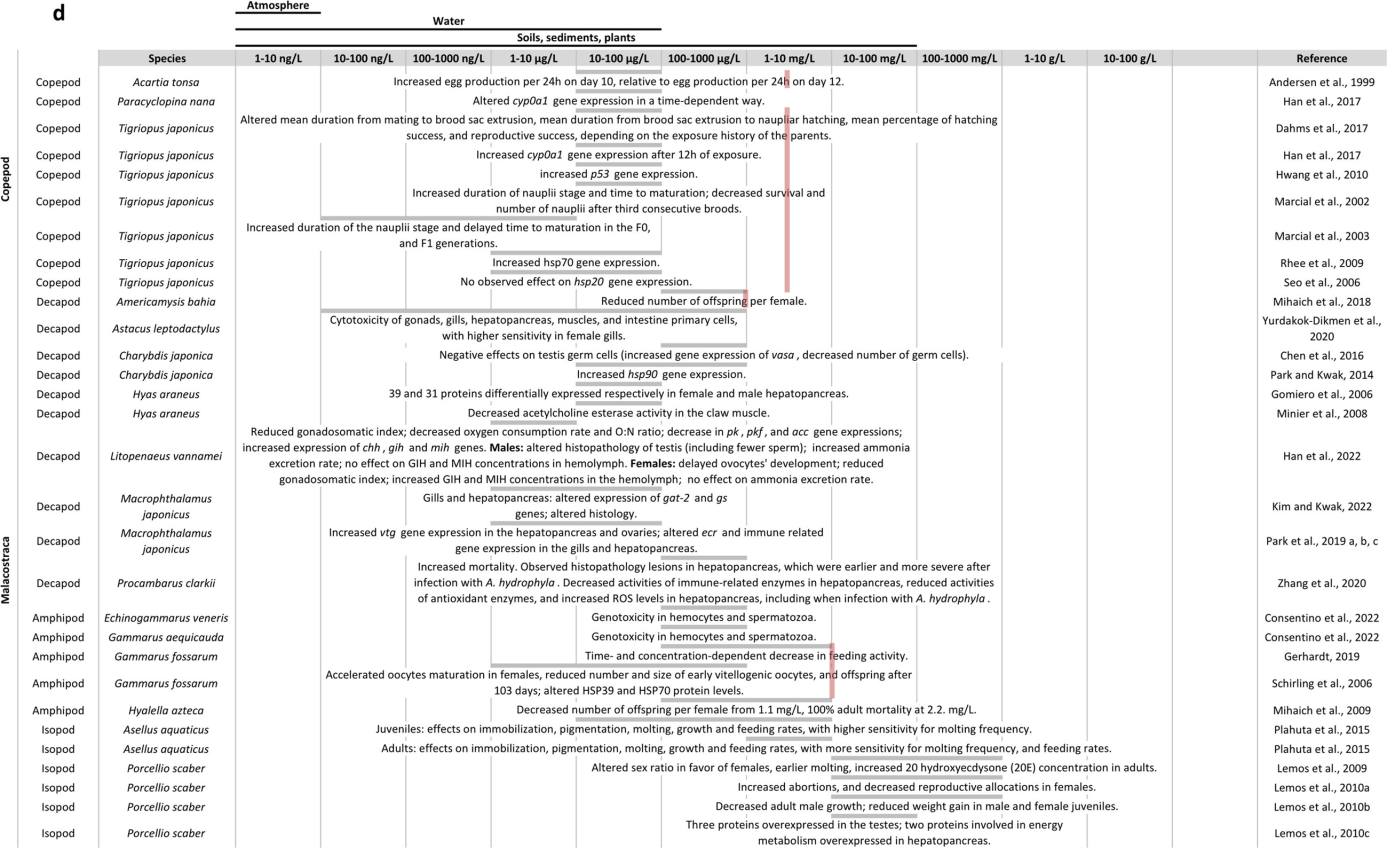
Overview of the literature on BPA effects in arthropods: **a**, **b** in insects, **c** in branchiopods, **d** in other arthropods. The table is organised with BPA concentrations in columns and arthropod species and references in rows. At each intersection, the effects found by the considered reference are described. For some species, a pink line is shown representing the mean observed LC50 at 24 h, 48 h or 96 h depending on current knowledge (Supplementary materials S2)

The effects of BPA exposure on arthropods are summarised in Fig. 2.

#### Toxicity, physiological and biochemical effects

BPA concentrations in the blue crab *Callinectes sapidus*, the shrimp *Macrobrachium rosenbergii* and the lobster *Homarus americanus* were in the Ranges of 0.10–0.44, 0.67–5.51 and 4.48–7.01 ng/g wet weight (ww), respectively (Zuo et al. 2015). In the prawn *Penaeus monodon* and in the crab *Portunus pelagicus*, mean BPA concentrations were respectively 13.3 ng/g ww and 213.1 ng/g ww (Basheer et al. 2004). A recent study also showed that, in the decapod *Litopenaeus vannamei*, concentrations of BPA were around 2 µg/L in ovaries and a bit below this value in testes (Han et al. 2022). There are fewer studies regarding BPA Internal concentrations In insects, but mean concentration in aerial insects was 5 ng/g (Park et al. 2009).

Regarding acute toxicity In Insects, 24 h LC50 in *Chironomus riparius* and *Chironomus tentans* were respectively, 6.030 mg/L (Ha and Choi 2008; Lee and Choi 2007) and 14.03 µM (around 3.265 mg/L) (Park and Choi 2007). When raised with food containing BPA, LC50 in *Drosophila melanogaster* was 12.35 mg/L of food (Anet et al. 2019). In the same species, 72 h LC50 was 211.349 mg/L In larvae and 269.153 mg/L in pupae (Begum et al. 2021), and 48 h LC50 In adult flies was 5.5 g/L (Musachio et al. 2021). Outside of Insects, In the order of decreasing sensitivity, 48 h LC50 were of 1.34 mg/L in *Americamysis bahia* (Hirano et al. 2004), 4.2 mg/L in *Acartia tonsa* (Andersen et al. 2001), 4.32 mg/L in *Tigriopus japonicus* (Marcial et al. 2002), 6.846 mg/L in *Diaphanosoma celebensis* (In et al. 2019), around 12.84 and 14.4 mg/L in *Daphnia magna* (Hirano et al. 2004; Nagato et al. 2016), 34.7 mg/L in *Artemia franciscana* (Castritsi-catharios et al. 2013) and 50.4 mg/L in *Artemia sinica* (Ali et al. 2014). 24 h LC50 in *Gammarus fossarum* was 10.60 mg/L (Gerhardt 2019), and 10 day LC50 in *Gammarus pulex* was 1.49 mg/L (Watts et al. 2001a).

BPA also affects detoxification, stress and antioxidative systems. In *Chironomus* sp., BPA induced genotoxic effects and expression of different hemoglobin gene isoforms (Ha and Choi 2008; Martínez-Paz et al. 2013; Park and Choi 2009), altered the expression of detoxification protein genes and/or activity of detoxification proteins (Cytochrome P450 4G—CYP4G, glutathione S transferase, catalase) and induced expression of *heat shock protein* (*hsp*) genes (*hsp27* and *hsp70*) (Lee and Choi 2007; Martínez-Paz et al. 2012; Watts et al. 2003). CYP4G subfamily catalyses the synthesis of cuticular hydrocarbons, involved in various biological functions like chemical communication and desiccation resistance (Feyereisen 2020). In *Drosophila melanogaster*, BPA at concentrations from 0.1 to 5 mg/L during post-embryonic development, and at 228 mg/L during embryonic development, increased oxidative stress (increased production of reactive oxygen species ROS and lipid peroxidation LPD), and reduced antioxidant defence system (reduction of superoxide dismutase SOD, and catalase CAT activities) (Anet et al. 2019; Musachio et al. 2021). Chronic exposure of flies to 0.0016 or 0.16 mg BPA/L can also affect the expression of genes involved in HSP (downregulation), metallothionein and stress signalling pathways (Frat et al. 2023). Besides, BPA exposure at 228 mg/L during embryonic development decreases cellular viability in *D. melanogaster* (Musachio et al. 2021). BPA also induced *hsp* gene expression in *Sesamia nonagrioides* (Michail et al. 2012). BPA exposure from 1 to 30 µg/L for 1 to 7 days affected the expression of hsp genes in *Macrophthalamus japonicus* (Park and Kwak 2019). In *Tigriopus japonicus*, exposure from 1 to 20 μg/L for 96 h increased hsp70 gene expression (Rhee et al. 2009), but exposure from 12.5 to 100 μg/L for 48 h did not affect hsp20 gene expression (Seo et al. 2006). BPA exposure also induced genotoxicity in hemocytes and sperm cells from *Echinogammarus veneris* and *Gammarus aequicauda*, for concentrations between 0.25 and 1 mg/L (Cosentino et al. 2022). In *Daphnia magna*, exposure to high concentrations (several mg/L) of BPA for 1 to 21 days affected the activity of antioxidant enzymes and the expression of *hsp70* and *hsp90* genes and genes associated with detoxification and antioxidation (Jemec et al. 2012; Kim et al. 2019; Li et al. 2018; Masteling et al. 2016). In the same species, 24-h exposure to environmental concentrations of BPA (some μg/L) induced DNA breaks, reduced catalase activity and increased mRNA expression of hemoglobin gene isoforms (Ha and Choi 2009; Park and Choi 2009).

In *Litopenaeus vannamei*, exposure to 2 µg/L BPA for 14 days resulted in reduced oxygen consumption rate, and decreased O:N ratio in males and females, and increased ammonia excretion rate in males (Han et al. 2022).

#### Development and metabolism

BPA exposure affects growth and development in arthropods. In the flies *D. melanogaster* and *Musca domestica*, BPA decreased pupae to larvae ratios, and affected development time (Atli and Ünlü, 2012; Izumi et al. 2008), growth rate, feeding (Weiner et al. 2014) and pupal weight (Izumi et al. 2008). Chronic exposure to 160 mg BPA/L in *D. melanogaster* significantly increased the duration of larval stage and delayed development (Frat et al. 2023). Exposure of late second Instar larvae for 72 h to 3.5 g/L in medium resulted in downregulation of genes involved in wing patterning (*i.e. wingless, decapentaplegic, vestigial*), and feeding, foraging and metabolic behaviour (*i.e. insulin-like receptor, foraging*) in *D. melanogaster* larvae and adults (Begum et al. 2021). BPA at 1 mM (i.e. 228 mg/L) reduced lifespan, decreased pupal eclosion rate and resulted in observed coloration and deformities in *D. melanogaster* pupae (Musachio et al. 2021). Raising *Culex quinquefasciatus Say* larvae In a medium with 1 to 4 mg/L BPA decreases time to pupation and to emergence, and significantly decreases pupal body weight at 26–31 °C (Valsala and Asirvadam 2022). In Lepidoptera, BPA affected larval and pupal stage durations (Kontogiannatos et al. 2015; Maria et al. 2019; Michail et al. 2012), decreased pupation success and percentage of emergence in *Sesamia nonagrioides* (Kontogiannatos et al. 2015) and affected pupal length in *Spodoptera littoralis* (Maria et al. 2019). In the ringed legged earwig, *Eurobellia annulipes*, Injections of 1.2 or 12 µg BPA for 6 days, altered duration of embryogenesis in offsprings (Rankin and Grosjean 2010). In *Chironomus riparius*, BPA induced moulting delays, reduced larval wet weight and delayed emergence (Martínez-Paz et al. 2013; Planelló et al. 2008; Watts et al. 2001b). In branchipods, BPA decreased body length in *Artemia franciscana* nauplii (Ekonomou et al. 2019) and *D. magna* (Chen et al. 2021), and interfered with pathways involved in development in *D. magna* (Fuertes et al. 2019). In copepods, 3-week exposure increased duration of *Tigriopus japonicus* nauplii stage in the F0 (from 0.1 µg/L), and F1 (from 0.01 µg/L) generations (Marcial et al. 2003), and altered moulting frequency (from 0.05 mg/L) and growth (from 0.5 mg/L) in *Asellus aquaticus* (Plahuta et al. 2015). In *Porcellio scaber*, exposure to higher doses (from 10 to 1000 mg/kg) for 2 and 16 weeks, respectively, affected growth in adult males and reduced weight gain in male and female juveniles (Lemos et al. 2010b). Little effects were observed on the development of *Acartia tonsa* (Andersen et al. 2001).

BPA affects carbohydrate and lipid metabolism. When raising *D. melanogaster* with 67.5 ng, 675 ng and 675 µg BPA/mg food media, Williams et al. (2014) observed an altered adult feeding behaviour and increased Lipid content after 24 h of starvation, and inhibition of insulin gene expression in Male flies. BPA exposure to 0.007 and 0.01 g/2 mL, for 30 generations in *D. melanogaster*, reduces larval foraging rate and foraging path length (Begum et al. 2020). Raising *Culex quinquefasciatus Say* larvae with 1 mg/L BPA induced the expression of the phospholipase A2 protein (Valsala and Asirvadam 2022). Injections of 0.12, 1.2 and 12 µg for 6 days reduced male weight gain in the ring legged earwig *Eurobellia annulipes* (Rankin and Grosjean 2010). Raising *Sesamia nonagrioides* with BPA affected larval weight (Michail et al. 2012). Exposure to 1.1 µg BPA/L from 4th larval Instar to day 6 of the last larval instar in *S. littoralis* significantly increased phosphoglucose isomerase activity (Rivas et al. 2023). In *Daphnia magna*, BPA exposure deregulated energy metabolism signaling pathways, increased Lipid droplet accumulation up to 95% (Fuertes et al. 2019) and altered feeding rates and increased fat storage in post-mating females (Jordao et al. 2016). Exposure between 1.03 and 10.3 mg/L BPA for 24 h in *D. magna* juveniles affected 20 metabolic pathways, mostly related with carbon metabolism (amino acid, urea, ammonia), and with glutamate playing a major role (Garreta-Lara et al. 2021). In *Porcellio scaber*, BPA concentrations from 10 to 1000 mg/kg of soil affected the levels of proteins involved in energy metabolism (Lemos et al. 2010a), and reduced weight gain in male and female juveniles (Lemos et al. 2010b). Exposure from 1 to 10 mg BPA/L inhibited feeding rate in *Asellus aquaticus* (Plahuta et al. 2015), and exposure from 1 to 3 mg BPA/L decreased feeding activity in *Gammarus fossarum* (Gerhardt 2019). In *Litopenaeus vannamei*, exposure to 2 µg/L BPA for 14 days decreased the expressions of genes involved in the glycolysis (pyruvate kinase and 6-phosphofructokinase-1) and metabolism of fatty acids (acetyl-CoA carboxylase) (Han et al. 2022).

#### Fecundity, hormonal pathways and immune system

Several studies showed effects of BPA on fecundity in arthropods. In *D. melanogaster*, exposure to BPA In food downregulated testis specific genes at 3.7 g/L (Branco and Lemos 2014), decreased mean fecundity, delayed mean Maturation times and decreased mean number of offsprings between 0.1 and 10 mg/L (Atli 2013; Atli and Ünlü, 2012). BPA exposure to environmental concentrations for several generations increased male/female ratio and decreased egg survival in *Musca domestica* (Izumi et al. 2008). In *Chironomus riparius*, exposure to BPA between 1 µg/L and 3 mg/L reduced yolk protein content in males and females (Hahn et al. 2002). Lower fecundity was observed in adult *Culex quinquefasciatus Say* raised with 2 to 4 mg/L BPA (reduced number of egg rafts and number of larvae hatched per egg raft) (Valsala and Asirvadam 2022). In *Eurobellia annulipes*, several days of exposure to BPA by injections (0.12, 1.2 and 12 μg) or in drinking water (from 0.1 to 100 μg/L) altered testis and seminal vesicle sizes, and gonadal growth (Rankin and Grosjean 2010). BPA in presence of Juvenile hormone III (JH III) increased the expression of *vtg* gene in the giant water bug *Lethocerus deyrollei* diapausing females (Nagaba et al. 2011). JH, which main form in insects is JHIII, is a terpenoid hormone, one of the two main lipidic hormones in insects with ecdysteroids. In particular, the interplay between JH and ecdysteroids is responsible for molt regulation in insects (Leyria et al 2022). Vitellogenin is a major lipoprotein and the precursor of yolk in egg-laying animals. BPA exposure induced the expression of vitellogenin in *Macrophthalamus japonicus* (Park et al. 2019a) and *Daphnia magna* (Kim et al. 2019). In the decapod *Charybdis japonicas*, BPA increased the gene expression of vasa, a protein widely used as a molecular marker for primordial germ cell and gametogenesis, and decreased the number of germ cells in the testis (Chen et al. 2016). In *Litopenaeus vannamei*, exposure to 2 µg/L BPA for 14 days resulted in reduced gonado somatic index in males and females, histopathology of testes and delay in oocytes’ developmental stages (Han et al. 2022). Three-week exposure to environmental concentrations of BPA delayed time to maturation in the F0 and F1 generations of *Tigriopus japonicus*, but induced no effect on fecundity, sex ratio or survival (Marcial et al. 2002). In the same species, 7 days of exposure to 0.1 mg/L affected reproductive success depending on the exposure history of the parents (Dahms et al. 2017). In *D. magna*, long-term exposure (21 days) to BPA from 3.45 to 13.8 mg/L decreased the number of neonates per adults (Jemec et al. 2012), but no effects were observed on the fecundity at concentrations up to 1 mg/L (Brennan et al. 2006), or on female body Length, clutch size or offspring size at up to 9 mg/L (Jordao et al. 2016). A more recent study found that exposure to 1.14 mg/L BPA for 21 days increased fecundity in the F0 generation of *D. magna* (Chen et al. 2021). However, Nguyen et al. (2020) found that exposure to 50 and 500 μg BPA/L for 10 days in *Ceriodaphnia cornuta* inhibited reproduction. In *Diaphanosoma celebensis*, exposure from 0.06 to 0.24 mg/L for 14 days delayed the emergence of the first offspring, and increased the cumulated number of neonates in a concentration-dependent way (Cho et al., 2022). In *Porcellio scaber*, 56 days of exposure to high concentrations increased abortion, affected sex ratio in favor of females and decreased reproductive females’ allocations (Lemos et al. 2010c). In *Gammarus fossarum*, exposure from 5 to 500 µg/L for up to 103 days accelerated oocyte maturation in females and reduced number and size of early vitellogenic oocytes (Schirling et al. 2006).

BPA has been observed to affect arthropods’ hormonal pathways. In *Chironomus riparius*, 1-day exposure to BPA induced the expression of estrogen-related receptor (ERR) gene (Park and Kwak 2010), and 3-h exposure increased *ecr* and *e74* gene expression in explanted salivary glands (Wang et al. 2019). E74 is an early response gene In the ecdysteroid pathway, encoding for a Transcription factor. Exposure from 1 to 4 mg/L BPA Induces earlier 20 hydroxyecdysone (20E) peak in fourth instar larvae in a concentration-dependent way (Valsala and Asirvadam 2022). In the same species, 1 mg BPA/L resulted in significant upregulation of *ecra* and *e75a* gene expressions in the last instar larvae (Valsala and Asirvadam 2022). In *Spodopdera littoralis*, Injection of 0.01 µg/g BPA decreased the hemolymphatic ecdysteroid peak at day 6 of last larval Instar, and Injection of 0.01 and 0.1 µg/g BPA affected the expression of ecdysteroid response genes in larval body and brain (Maria et al. 2019). Chronic exposure to *D. melanogaster* to 0.0016 or 0.16 mg BPA/L altered the expression of genes involved in hormone signalling pathways (upregulation of BR–C at 0.0016 mg/L, downregulation of cyp18a1 at 0.0016 mg/L and jheh1 at 0.16 mg/L) (Frat et al. 2023). One-day exposure to BPA (from 1 to 30 μg/L) increased *ecr* gene expression in *Macrophthalamus japonicus* gills and hepatopancreas (Park et al. 2019b), and exposure from 0.2 to 5 mg/L decreased *ecr* gene expression in *D. magna*, after 48 h but not after 24 h of exposure (Kim et al. 2019). In *D. magna*, exposure to 1.14 mg/L BPA for 21 days inhibited the expression of genes involved in endocrine pathways: cytochrome P450 314 (*cyp314)*, ecdysteroid receptor a and b (*ecra* and *ecrb*), ultraspiracle (*usp*), and vitellogenin 1 and 2 (*vtg1* and *vtg2*) in the F0 generation (Chen et al. 2021). Cyp314 is responsible for the synthesis of the molting hormone. Exposure to high concentration of BPA (10 mg/L) enhanced the effect of methyl farnesoate (10 µg/L) by increasing the number of broods containing males in *D. magna* (Jeong et al. 2013; Wang et al. 2005). Methyl farnesoate is a terpenoid hormone found in *D. magna* and other crustacean taxa, with a similar structure and function as Juvenile hormone in insects, in particular in favoring maintenance of juvenile characters. In *Diaphanosoma celebensis*, 48-h exposure to BPA altered the expression of genes involved in the ecdysteroid pathway: *ecr* and *usp* gene expression was found to be Increased at 0.6 mg/L in In et al. (2019) but not in (Cho et al. 2022); conversely, decreased expression of those genes was observed at 0.12 and 3 mg/L in (Cho et al. 2022) but not in In et al. (2019). Increased expression of genes Involved in the JH pathway was also observed at 0.6 mg/L (Cho et al., 2022). In an in vitro competition study with *Homarus americanus* EcR, the concentration of BPA reducing the binding of labelled ponasterone A by 50% was 0.16 mM, showing that BPA had low affinity for this receptor (Tarrant et al. 2011). Exposure to 2 µg/L BPA for 14 days in *Litopenaeus vannamei* increased the expression of *crustacean hyperglycemic hormone (chh)*, *gonad inhibiting hormone (gih)* and molt inhibiting hormone (*mih)* in both males and females, and increased GIH and MIH concentrations in female hemolymph (but not in males) (Han et al. 2022). The CHH hormones are divided in two families: (1) the CHH, involved in carbohydrate metabolism regulation, and (2) the inhibitory hormones like GIH (also called Vitellogenesis-Inhibiting Hormone (VIH)) and MIH, which inhibit vitellogenesis and molt respectively.

Exposure to 1, 10 and 30 µg/L BPA affected the expression of immune-related genes in *Macrophthalamus japonicus* (Increase after 1 day but decrease at 4 and 7 days) (Park and Kwak 2019).

In *M. japonicus*, histopathology of gills and hepatopancreas was observed after exposure to 1 to 30 µg/L BPA for 1 to 7 days (Kim and Kwak 2022).

#### Nervous system and behaviour

Most studies on BPA effects on the nervous system and behaviour in arthropods are recent. BPA affects locomotory and social behaviour in *D. melanogaster*, where exposure from 0.001 to 1 mM (approximately 0.228 to 228 mg/L) increased the number and duration of grooming episodes, decreased distance with their closest neighbour in a social setting, and induced changes in exploratory behaviour (Kaur et al. 2015; Musachio et al. 2021). Exposure to 1 mM BPA also decreased dopamine level and tyrosine hydroxylase activity, but induced no significant effect on tyrosine level (Musachio et al. 2021). Seventy-two-hour exposure to 3.5 g/L BPA in *D. melanogaster* larvae induced a general decrease in observed courtship behavioural features, especially when the male were exposed. Besides, *doublesex* and *fruitless* genes were upregulated in adult males and downregulated in adult females (Begum et al. 2021). In the decapod *Hyas araneus*, exposure to 50 µg/L BPA for 3 weeks decreased acetylcholine esterase activity in the claw muscle (Minier et al. 2008). In a multigenerational study, Begum et al. (2020) showed that larvae from the 30th generation had reduced feeding rates and foraging paths, and adults from the 31 st generation displayed an altered ability to climb, and a reduced duration for the investigated courtship items. In *M. japonicus*, exposure from 1 to 30 µg/L BPA for 1 to 7 days alters the expression of genes involved in neurotransmission (gamma-aminobutyric acid Transporter Subtype 2, and glutamine synthetase) in gills and hepatopancreas (Kim and Kwak 2022). In *D. magna*, exposure from 0.4 to 1.8 mg/L for 48 h in juveniles results in decreased expression of the slc6a1 gene, involved in signal transduction, and alters behaviour: decreased activity during light intervals, but increased activity during dark intervals (Chen et al. 2021). BPA exposure for 21 days also decreased swimming speed in the F0 (11.4 µg/L BPA) and F1 (1.14 mg/L) generations (Chen et al. 2021). In the water louse *Asellus aquaticus*, exposure to BPA in water, sediment of food, resulted in reduced feeding rate and mobility in adults and juveniles (Plahuta et al. 2015). The reproductive behaviour of *Gammarus pulex* was affected only by high concentration of BPA (8.4 mg/L), suggesting that this was not due to endocrine disruption (Watts et al. 2001a).

#### Conclusions on BPA effects in arthropods

BPA was found in arthropods in concentrations Ranging from 0.10 to 214 ng BPA/g ww. Regarding acute toxicity, most studies on LC50 were done In aquatic environment. BPA 48 h LC50 Ranged between 1.34 mg/L for *Americamysis bahia* and 50.4 mg/L in *Artemia sinica*. 24 h LC50 was slightly lower in *Chironomus* sp. (between 3 and 7 mg/L) than in *Gammarus fossarum* (10.60 mg/L). In *D. melanogaster* exposed via food, 48 h LC50 was 5.5 g/L. The observed genotoxic effects of BPA in *Chironomus* sp. and *Daphnia magna* may be induced by oxidative stress. Indeed, BPA exposure increased oxidative stress and reduced antioxidant defence system in several arthropod species. BPA affects growth and post-embryonic development in several species. In particular, BPA-induced effects on development time and decreased pupation or emergence successes were observed in *D. melanogaster*, *Culex quinquefasciatus*, *Sesamia nonagrioides*, and in copepods *T. japonicus* and *Asellus aquaticus*, which may be related to the effects of BPA on carbohydrate and lipid metabolism. Indeed, BPA was observed to affect many metabolism-related endpoints: altered feeding behaviour, increased lipid content and insulin gene expression in *D. melanogaster*; increased Lipid droplets, altered feeding rates and altered metabolite Levels from 20 metabolic pathways in *D. magna*; and decreased expression of genes involved in the glycolysis and fatty acid metabolism in *Litopenaeus vannamei*. BPA affected feeding rates in *D. melanogaster*, *D. magna*, *Asellus aquaticus* and *Gammarus fossarum*, and altered larval or adult weights in *Eurobellia annulipes*, *Sesamia nonagrioides and Porcelio scaber*. Fecundity and reproduction were also affected by BPA in *D. melanogaster*, *Musca domestica*, *Chironomus riparius*, *Culex quinquefasciatus* and *Eurobellia annulipes*. This may be due to BPA effects on energy metabolism, and/or on hormonal pathways. Indeed, BPA affects the ecdysteroid pathway in *C. riparius*, *D. melanogaster*, *M. japonicus* and *D. magna*; the juvenile hormone pathway in *Lethocerus devrollei*; and CHH hormone levels in *Litopenaeus vannamei*. Two studies found effects of BPA on immune-related genes in *M. japonicus*. Most studies investigating BPA effects on the nervous system and behaviour were done on *D. melanogaster*. As compared to DEHP, most studies on BPA focus on mobility or swimming behaviour instead of sexual behaviour. While there are more articles on BPA effects on arthropods than for DEHP, BPA mechanisms of actions in arthropods are still unknown and are necessary to interpret the results of those studies.

## Research gaps and areas of investigation

With the massive use of plastics worldwide, plastic chemicals like BPA and phthalates are ubiquitous in the environment. Many studies have underlined the detrimental endocrine disrupting effects of those chemicals in vertebrates, but their effects on arthropods have been much less studied. While arthropods represent a major part of animal diversity and play a critical role in both terrestrial and aquatic ecosystems, many research gaps remain today as to the consequences of plastic chemicals’ exposure on arthropods.

### Are BPA and DEHP endocrine-disrupting chemicals in arthropods?

Endocrine-disrupting chemicals (EDCs) are one of todays’ major issues regarding human and environmental health. A widely recognised definition of an EDC is “an exogenous substance or mixture that alters function(s) of the endocrine system and consequently causes adverse health effects in an intact organism, or its progeny, or (sub)populations” (WHO 2012). Due to their action on hormonal signalling pathways, EDCs have properties that make them particularly dangerous and difficult to regulate. Most studies have been done on mammals and have shown that EDCs can act at low doses, induce non-monotonous dose-responses, have specific windows of action during which they can be particularly dangerous (i.e. during pre-natal and post-natal life and puberty) and can induce delayed or even inter-generational effects (Baines et al. 2017). The possible underlying mechanisms of actions explaining the ability of EDCs to induce non-monotonous dose response curves have been reviewed (Gore et al. 2015; Kortenkamp et al. 2009; Matthiessen et al. 2018; Trasande et al. 2015). Different mechanisms have also been proposed to explain delayed and inter-generational effects of EDCs (Chen et al. 2015; Manikkam et al. 2013; Verdoux et al. 2017), in particular (1) changes in hormonal levels during development resulting in later consequences in adults or progeny—Developmental Origins of Health and Disease (DOHaD) (Chen et al. 2015; Manikkam et al. 2013; Verdoux et al. 2017); and (2) the involvement of epigenetic mechanisms, i.e. a regulation of gene expression unrelated to DNA sequence, and that could be transmitted through mitosis and meiosis (Rissman and Adli 2014; Vandegehuchte and Janssen 2014; Walker and Gore 2017).

In this review (Fig. 1; supplementary data S2), we show that several studies found effects of DEHP on endpoints under hormonal regulation like growth and development (e.g. Frat et al. 2023 and Dao et al. 2022), and reproduction and fecundity (e.g. Green-Ojo et al. 2024a, Cuvillier-Hot et al., 2024). But these endpoints can also be affected by other biological mechanisms than hormones, such as metabolic resources. DEHP was also found to affect the expression of the ecdysteroid receptor (*ecr*) gene in *Chironomus riparius* (Planello et al., 2011, Herrero et al. 2017), *Spodoptera littoralis* (Aviles et al. 2019, 2020) and *Macrophthalamus japonicus* (Park et al. 2019a). However, these studies have used different ways of exposure (i.e. in water or in the food), different concentrations, and were performed either in larvae or in adults and, therefore, found different results as to an increase or decrease of *ecr* gene expression. There were generally more articles on the effects of BPA in arthropods. Several also show an effect of BPA on endocrine-related endpoints like growth and development (e.g. Nguyen et al. 2020; Rivas et al. 2023) and reproduction and fecundity (e.g. Rankin and Grosjean 2010; Cho et al., 2022). In branchiopods, BPA was generally found to decrease the expression of genes involved in the ecdysteroïd pathway (Kim et al. 2019 and Cho et al., 2022) and increase the expression of genes involved in the JH pathway (Cho et al., 2022). In hexapods, exposure to BPA generally increased the expression of ecr gene (Planello et al. 2008, Wang et al. 2019, Valsala and Asirvadam 2022, and Kontogiannatos et al. 2015), and induced an increase in JHII and JHIII levels in *Musca domestica* (Izumi et al. 2008). In malacostraca, BPA would affect the expression of ecr gene in *Macrophthalmus japonicus* (Park et al. 2019a), the expression of Gonad Inhibitory Hormone (gih) and Molt Inhibitory Hormone (mih) genes and levels of GIH and MIH in *Litopenaeus vannamei* (Han et al. 2022), and Increase the concentration of 20E in *Porcellio scaber* (Lemos et al. 2009). Although articles are more numerous than for DEHP, the diversity of species, concentrations, time of exposure and developmental stages used in these studies make it difficult to draw conclusions as to a possible mechanism of action of BPA in arthropods. Besides, as discussed earlier in this review, BPA was shown to have low affinity for *Homarus americanus* EcR (Tarrant et al. 2011), which suggests that the observed effects of BPA on endocrine endpoints does not result from its binding to EcR.

**Fig. 3:**
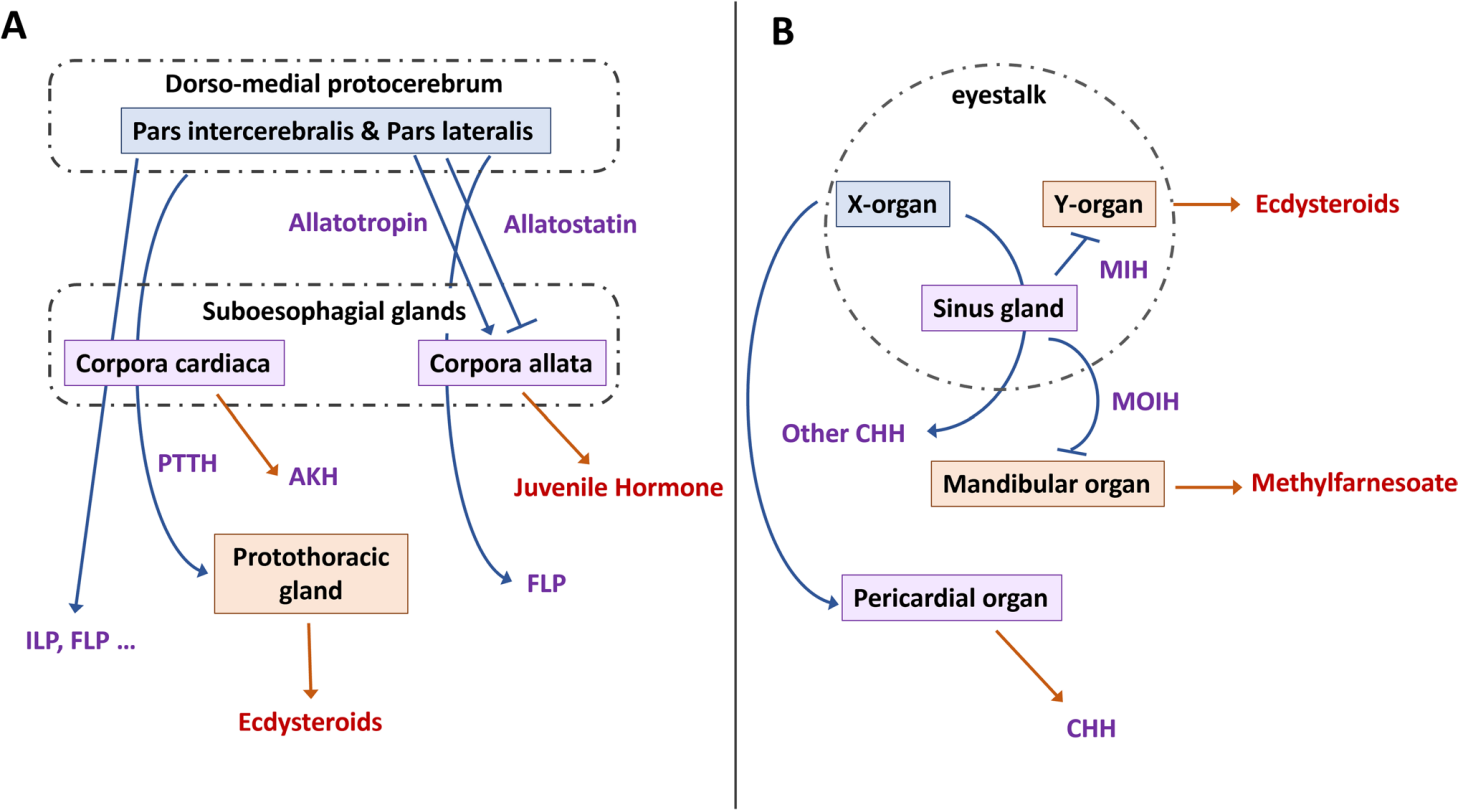
Schematic representation of neurohormonal system in arthropods: **A** in insects,** B** in decapods. Blue box: neuroendocrine centre; purple box: neurohemal organ; orange box: gland; blue arrow: production of neurohormones; orange arrow: production of hormones; purple text: neurohormones; red text: lipidic hormones. AKH, adipokinetic hormone; CHH, crustacean hyperglycemic hormone; FLP, FMRFamide-like peptide; ILP, insulin-like peptide; MIH, molt-inhibiting hormone; MOIH, mandibular organ–inhibiting hormone; PTTH, prothoracicotropic hormone

**Fig. 4:**
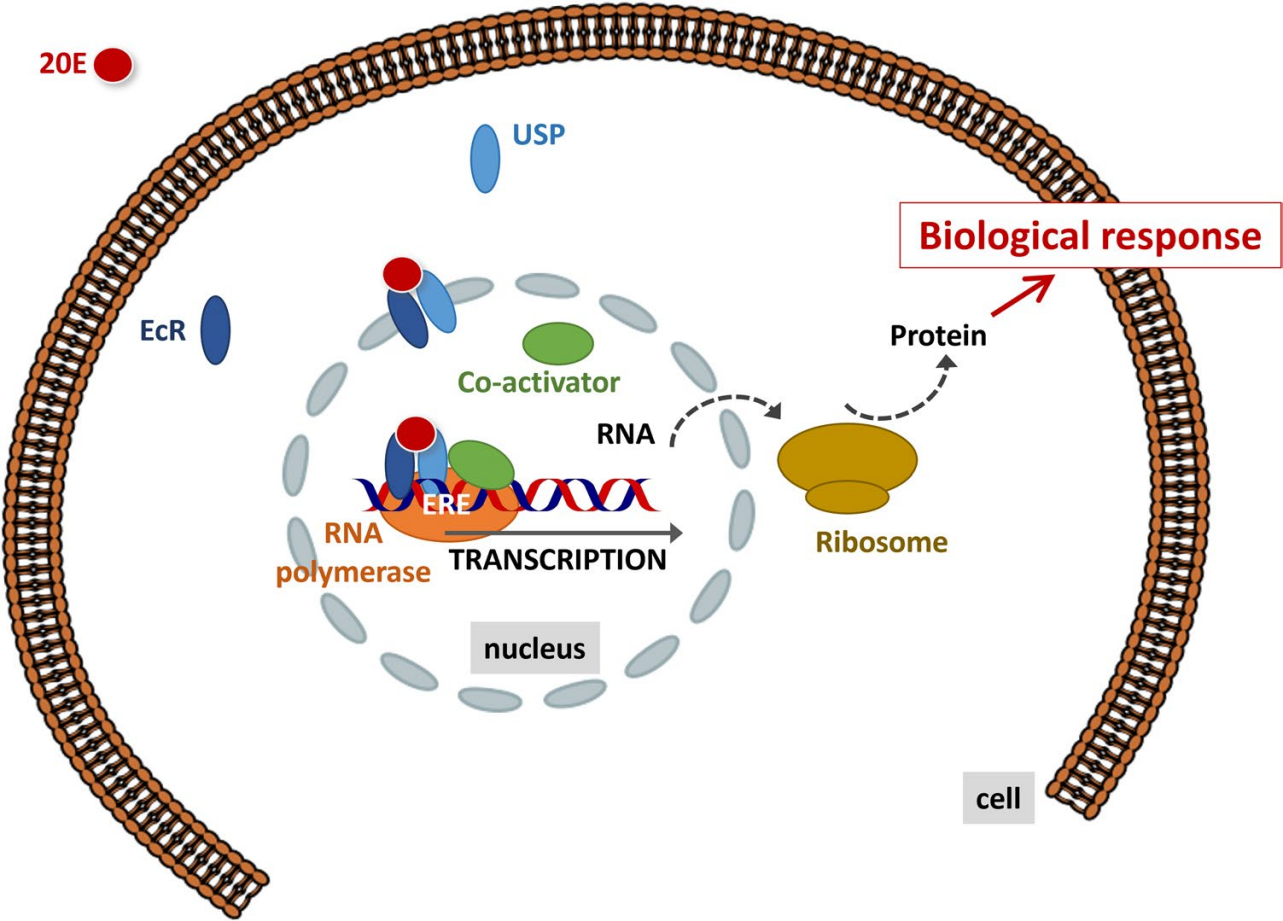
An example of 20 hydroxyecdysone (20E) mechanism of action In Insects: 20E enters the cell and interacts with the ecdysteroid receptor (EcR) and ultraspiracle (USP) to form a functional ecdysteroid receptor. The complex then migrates inside the nucleus and interact with the ecdysteroid response element (ERE) of the DNA. The fixation of the RNA polymerase and co-activators enables the transcription of the DNA to RNA. The RNA then migrates in the cytoplasm to initiate the biological response, in particular through the translation of the RNA to a protein

**Fig. 5:**
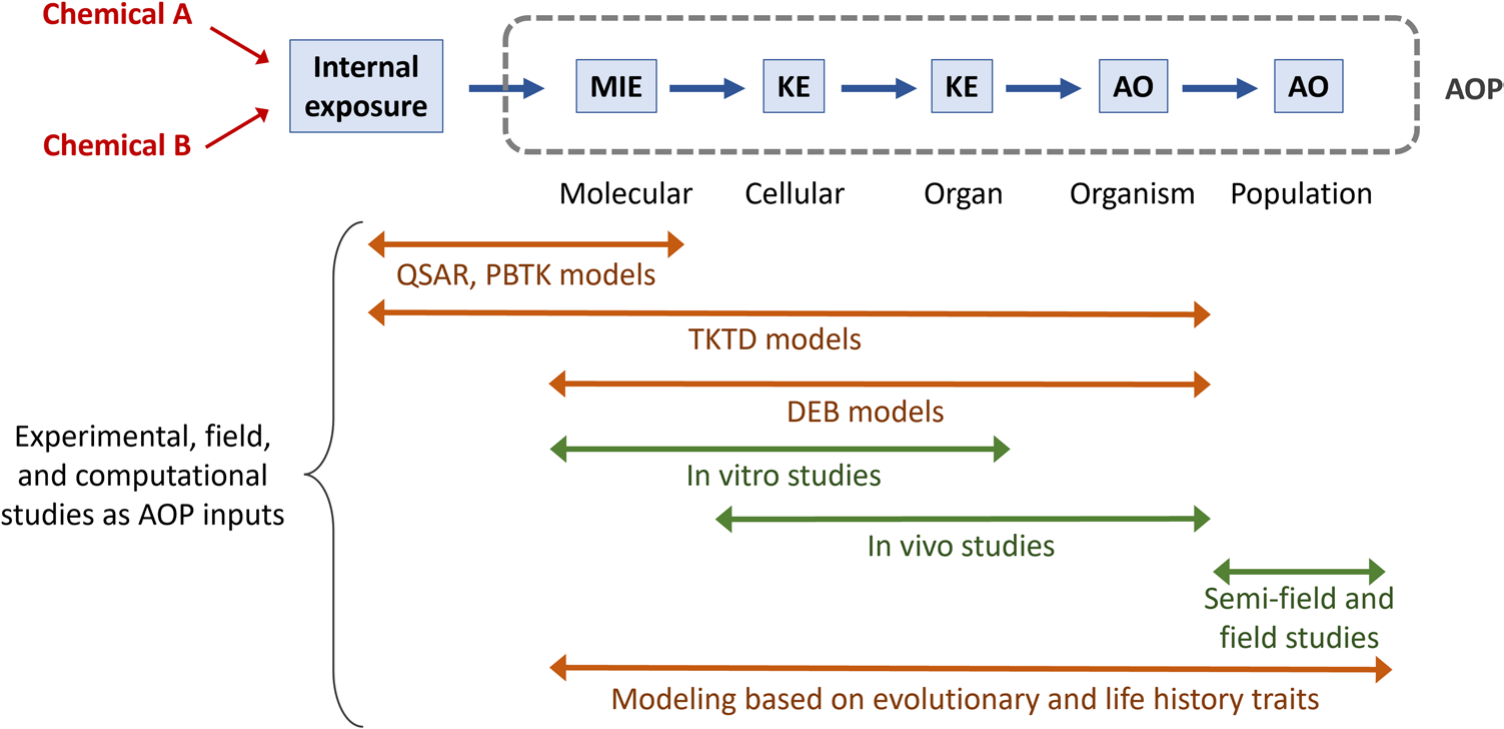
Schematic representation of the adverse outcome pathway (AOP) concept. MIE, molecular initiating event; KE, key event; AO, adverse outcome; QSAR, quantitative structure activity relationship; PBTK, physiologically based toxicokinetic; TKTD, toxicokinetic toxicodynamic; DEB, dynamic energy budget. Orange arrows and text represent computational tools, green arrows and text represent experimental and field studies

In a recent article, Crane et al. (2022) review the knowledge and tools available to assess the endocrine disrupting effects of chemicals on invertebrates in the European Union. The authors specify that for a chemical to be considered an EDC in an (intact) organism, the adverse effect(s) observed after exposure should result from an endocrine mode of action. There are two main obstacles to the identification of EDC in invertebrates: (1) the breadth of invertebrate endocrine systems (due to the wide diversity amongst invertebrates, and to the diversity of endocrine pathways in each taxon); and (2) the lack of knowledge of invertebrate endocrine systems, even in arthropods, whose endocrine systems have been more studied. Figure 3 provides a schematic representation of the neurohormonal system in arthropods, and Fig. 4 an example of 20E mechanism of action in insects. Furthermore, the authors state that “there are no validated tools to determine any invertebrate endocrine mode of action in vitro or in vivo”. Indeed, only a few OECD guidelines are available on invertebrate, and none of them, together or separately, make it possible to identify an endocrine mode of action. There is a growing consensus in the regulatory and (eco)toxicological communities that Adverse Outcome Pathways (AOP) framework can be useful to put together in silico, in vitro, in vivo data and population modelling. In the AOP framework (Fig. 5), a Molecular Initial Event (MIE) induces successive Key Events (KE), linked by Key Event Relationship (KER) to result in an Adverse Outcome (AO) at organismal or population level (Ankley et al. 2010). Using the AOP framework an EDC can be defined as a chemical that has an endocrine mechanism (i.e. a MIE part of an endocrine pathway) causally linked via KEs to an AO. Only three AOPs were identified to be relevant for invertebrate endocrine disruption (Crane et al. 2022), two focusing on the juvenile hormone and the ecdysteroid pathways, with applicability on *Daphnia* and one on 5-Hydroxytryptamine transporter (5-HTT) with applicability to molluscs.

The World Health Organisation (WHO) definition of an EDC is species specific. So far, most studies on endocrine disruption in arthropods used chemicals recognised as EDCs in humans or other vertebrates, like DEHP and BPA, with the assumption that they would also have an endocrine disrupting effect in arthropods. This issue has been discussed in more details by Knigge et al. (2021). In their paper “A Crab is Not a Fish”, the authors point out the fact that deuterostome (containing the vertebrates) and protostome (containing the arthropods) have diverged more than 500 million years ago, hence leading to the development of unique endocrine pathways in arthropods, different from the endocrine system in vertebrates. Despite several studies showing effects of DEHP and BPA on endocrine endpoints in arthropods, the mechanisms of action of those chemicals in arthropods remain mostly unknown. Given the pleiotropic effects of DEHP and BPA in a wide range of species, it is likely that, as in vertebrates, they act through different mechanisms of actions. One further difficulty to determine whether DEHP and BPA are EDCs in arthropods is the stronger inter-connection between the nervous and hormonal system in arthropods (deFur 2004). It can be therefore expected that a chemical acting on the central nervous system could indirectly affect the hormonal system, e.g. during metamorphosis. Similarly, DEHP and BPA seem to have a strong effect on lipid and carbohydrate metabolism, which could indirectly affect hormonal endpoints.

To conclude, determining the mechanisms of action of DEHP and BPA is critical to categorise those plastic compounds as EDCs in arthropods. While DEHP and BPA are two of the main studied chemicals in arthropods, and despite their observed effects on endocrine-relevant endpoints, the available data described here are insufficient to determine if they are endocrine-disrupting chemicals in arthropods.

Nevertheless, those chemicals exert various effects on arthropods at environmentally relevant concentrations, and could have critical consequences for arthropod populations and the environment, therefore calling for further research in this area. Putting aside the concept of EDC, it is essential to gather further knowledge on invertebrates’ endocrine and nervous systems, which both play a critical role in adaptation to internal and external factors, and in particular on the roles and mechanisms of actions of hormones and neuromodulators as regards plasticity (Rissman and Adli 2014; Vandegehuchte and Janssen 2014; Walker and Gore 2017).

### Gaining more insight on how arthropods’ behaviours are regulated, and increasing the use of behavioural studies in arthropods’ ecotoxicology

A better understanding of the effects of chemicals on behaviour would require research on the role of hormones on modulation of arthropods’ behaviour (Bear et al. 2017; Mathiron et al. 2019), the assessment of behaviour in the frame of ecotoxicology (Bear et al. 2017; Mathiron et al. 2019) and the possible consequences for arthropod populations of altered behaviour due to chemical exposure (Kohler et al. 2018; Pyle and Ford 2017).

**Fig. 6:**
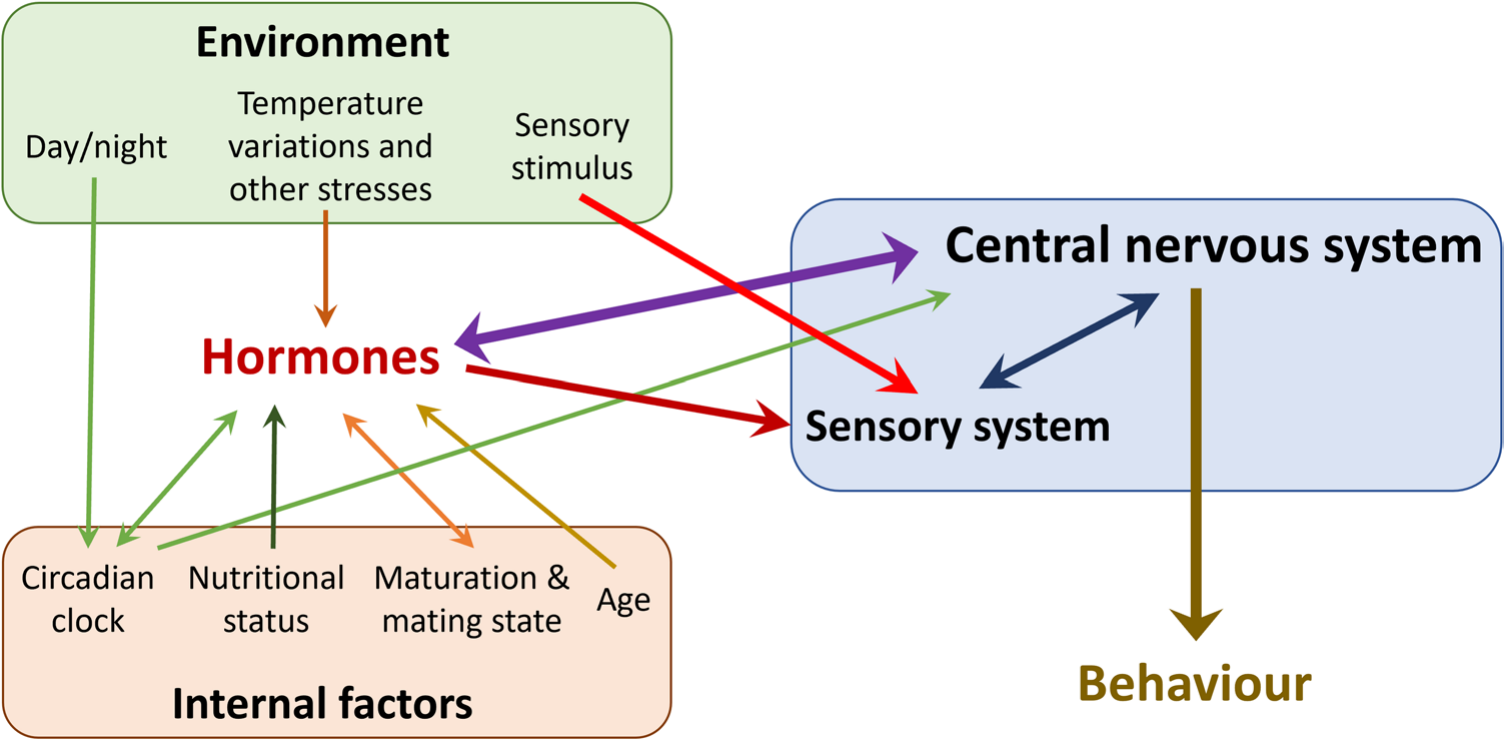
Summary of factors influencing behavioural response to a sensory stimulus

As in vertebrates, behaviour is under the control of the nervous system in arthropods (Bicker and Menzel 1989). In some cases, behaviours can be triggered by sensory stimuli, as the olfactory-induced sexual behaviour in the cotton leafworm *Spodoptera littoralis* (Aviles et al. 2020). Beside nervous regulation, behaviour is also under hormonal regulation (Fig. 6). For example, JH has been found to be involved in the number of mature eggs in female wasps *Eupelmus vuilleti* and their aggressive behaviour (Mathiron et al. 2019). Several studies also show that JH is involved in division of labour and aggressiveness in several species like ants (Norman and Hughes 2016) and the wasp *Polistes dominulus* (Tibbetts and Izzo 2009). Similarly, ecdysteroids can affect *Homarus americanus* agonistic behaviour in a sex-specific way (Reinhart et al. 2012).

Behaviour plays critical roles in arthropods’ population such as (1) agonistic interactions in decapods (Aquiloni et al. 2012; Coglianese et al. 2008; Reinhart et al. 2012); (2) division of labour and foraging in social insects, thus affecting social organisation of those populations (Mathiron et al. 2019; Norman and Hughes 2016; Tibbetts and Izzo 2009); and (3) reproduction and mating (Bigot et al. 2012). By interfering with those behaviours, plastic chemicals can thus interfere with arthropods’ fitness and populations’ dynamics.

Behaviour provides a good link between environmental contamination and the response of the organism at the molecular and physiological levels, and has consequently become a major endpoint in environmental toxicology (Anton et al. 2011). Behaviour is a key organisational scale to bridge the gap between mechanistic laboratory experiments and environmentally relevant field studies, and better understand the consequences of exposure to pollutants at the population and community levels. Behaviour is also a very sensitive endpoint, as it is generally disturbed at lower exposure levels than other conventional endpoints like development, reproduction and mortality (Bertram et al. 2022).

Despite the expanding field of behavioural ecotoxicology, still few studies have investigated the effects of chemicals on arthropods’ behaviour. One good example on this matter are a series of articles focussing on the effects of antidepressants on amphipods’ phototactic behaviours and anxiety, showing that, as the serotonin, dopamine and norepinephrine systems are well conserved, behaviour is a sensitive endpoint to study the effects those chemicals in arthropods (Bossus et al. 2014; De Castro-Català et al. 2017; Fong and Ford 2014; Guler and Ford 2010; Perrot-Minnot et al. 2017). From a methodological point of view, behavioural studies are subjected to a lack of repeatability. For example, by studying the effects of size and shape of the arena on amphipods’ behaviour, the size of the arena was observed to play a critical role in behavioural endpoints, suggesting that too small an arena would not provide the space for amphipods (and more generally other animals) to “behave” and thus results in false negative results (Kohler et al. 2018).

In this review, seven studies investigated the effects of BPA on arthropods’ behaviour. Two were done on *Daphnia magna* and found that BPA decreased swimming speed (Chen et al. 2021) along with cumulative distance and activity (Yuan et al. 2021). Four studies were done on *Drosophila melanogaster*, and found that BPA exposure altered climbing ability (Begum et al. 2020), decreased duration or occurrences of courtship behavioural features (Begum et al. 2021), increased number and duration of grooming episode and affected exploratory behaviour and social interaction (Kaur et al. 2015; Musachio et al. 2021). The last study Investigated the concentration of BPA that Induced an effect on locomotor activity on 50% of the individuals (EC50) on several species (Gerhardt et al. 2019). Only five studies assessed the effect of DEHP on arthropod behaviour. Knowles et al. (1987) observed an effect of DEHP on surfacing behaviour of *Daphnia magna*. Exposure to DEHP also affected the ability of dragonfly larvae to catch preys (Woin and Larsson 1987). In *Drosophila melanogaster*, exposure to DEHP affected copulation duration in a sex-specific way (Chen et al. 2019). Exposure to DEHP altered the frequency and duration of behavioural items in *Spodoptera littoralis* Males at 676 ng/g of food, and increased the latency of mating (Aviles et al. 2020). A more recent study on *Echinogammarus marinus* found that DEHP did not have significant effects on swimming behaviour at concentrations Ranging from 0.5 to 500 μg/L (Green-Ojo et al. 2024b).

Bertram et al. (2022) published a very good review on the advances and challenges in the field of behavioural ecotoxicology. They argue for increasing environmental realism, ecological complexity and mechanistic understanding in behavioural ecotoxicology (Bertram et al. 2022). In particular, experimental design should consider behaviours that are ecologically relevant to the considered species and biologically targeted by the studied contaminant. The experimental design should also take into account the natural social environment of the species, the inter-individual behavioural variations that may reflect phenotypic plasticity, circadian/circannual rhythms or life-history events, and include a multi-stressor approach (Bertram et al. 2022).

Historically, in chemical risk assessment, ecotoxicological experiments should follow strict guidelines and straightforward statistical analyses (Bertram et al. 2002) that have been set to standardise the procedure and increase validity of the experiments. It is also assumed that reliability and reproducibility depend on reducing internal variability through “environmental standardisation” (Peterson et al. 2017). However, this approach, instead of increasing reliability, could result in increasing the effect of possible confounding factors on the results (Richter and von Kortzfleisch 2020; Voelkl et al. 2020). Besides, the concept of “environmental standardisation” is in contradiction with the need to increase environmental realism and ecological complexity of behavioural ecotoxicology experiments, discussed in Bertram et al. (2022).

### Better understanding arthropods’ epigenetics, the effects of plastic chemicals on those mechanisms and the possible consequences for populations

Many researches have underlined the possible action of some chemicals on epigenetic mechanisms, in particular DEHP and BPA (Singh and Li 2012). Epigenetic mechanisms induce long-term changes in gene expression, and are therefore considered to be a possible mechanism underlying long-term and transgenerational effects (Chen et al. 2019; Martínez-Guitarte et al. 2012).

Studies on arthropods showed that epigenetic mechanisms are involved in division of labour in eusocial insects (Rissman and Adli 2014), and in cast determination (e.g. workers or queens) in honey bees (Herb 2014; Yan et al. 2014). Epigenetic inheritance observed in rodents (Vandegehuchte and Janssen 2014) can also occur in insects (Manikkam et al. 2013), possibly inducing long-term effects on insect populations. For instance, after exposure to heat shock for two generations in the fly *Drosophila melanogaster*, the transcription-factor dependent disruption of heterochromatin formation is transmitted epigenetically to following generations, even in the absence of the initial stress (Stern et al. 2012). Furthermore, epigenetics is considered a possible mechanism of adaptation in arthropods. In *Daphnia magna*, for example, sex determination and adult morphology is epigenetically determined (Stern et al. 2012). Epigenetics is also believed to be involved in brain and behavioural plasticity to environmental cues (Robichaud et al. 2012), and is suggested to take part in insect pesticide resistance.

Given the critical role of epigenetics in regulating different biological processes, including in the next generations, a better understanding of how DEHP and BPA could interfere with arthropod’s epigenetic mechanisms would also contribute to the assessment of their effects on arthropod populations and the possible evolutionary consequences on a long-term scale. For example: To what extend could arthropods adapt to those chemicals? What are the differences between species on this regard? How could it affect the relative abundance of each species and the ecosystem as a whole? (Singh and Li 2012). More generally, Vandegehuchte and Janssen (2014) argued that ecotoxicology still lacks an evolutionary perspective, which would increase our ability to predict the consequences of contaminant exposures on populations and higher levels of biological organisation (i.e. communities and ecosystems) (Brady et al. 2017). Besides, if we observe the changes in response to chemicals along a phylogenetic tree, we could better predict possible effects for non-model species, therefore gaining a better understanding of the effects of chemicals at the ecosystem level (Peterson et al. 2017).

### Including the effects on arthropods’ microbiota in ecotoxicological studies

The use of omics technologies has resulted in the discovery of the critical and multiple roles of microbiota within host organisms, which has been previously hampered by the inability to cultivate most microbial species. It is now known that microbiota can be involved in numerous biological functions like nutrition, development, immunity and behaviour, and it is assumed that most animals host microbiota. In human, for example, microbiota represents as Many cells as Human cells, and up to 1000 times more genes, hence questioning the limits of individual species (Duperron et al. 2020). This has led to the development of the “holobiont” concept that comprises both the studied organism and the hosted microbiota. Most of the time, microbiota is associated with epithelia, at the frontier between the host and external environment. Microbiota can therefore represent a first line of defence against chemicals or other stresses, but its composition or activity may also be affected by those stresses (Duperron et al. 2020).

Most studies on microbiota focus on microbiota composition, mostly bacterial microbiota due to the availability of a well-established technique: amplicon sequencing with 16S rRNA gene as a target (Duperron et al. 2020). However, microbiota composition does not always reflect microbiota functionalities, as some closely related bacteria may have very different functions (e.g. *Vibrios*). Besides, using amplicon sequencing to investigate bacterial microbiota, results in leaving apart other microorganisms like protists, fungi, archaea and viruses, which also play a critical role within the microbial community. Another limit of amplicon sequencing is that it results only on relative quantification of bacterial communities, while absolute quantification, for example using quantitative PCR would be more informative, and enable comparisons between studies (Duperron et al. 2020).

Antonelli et al. (2022) reviewed the interactions between anthropogenic stressors, and insect microbiota. Insect microbiota can directly contribute to detoxification through metabolisation of the compound, sequestration, bacterial efflux pumps or the formation of a protective biofilm preventing the toxic compound to enter insect cells. Microbiota can also contribute indirectly to detoxification, through interaction with its host, for example, by triggering an increased expression of insect detoxification genes. In some cases, insect adaptation to anthropogenic stressors occurred through the integration of a new endosymbiont (Antonelli et al. 2022). Insect’s microbiota response to xenobiotics is highly dependent on abiotic (e.g. Temperature, other xenobiotics in the environment) and biotic (e.g. composition of the microbiota) factors, with high inter-individual variability within insects. Besides, available studies are biased towards insect models of relevance for health of agriculture, and widely used xenobiotics (Antonelli et al. 2022). Thus, to our knowledge, no study has been published so far on the effect of DEHP or BPA on insect microbiota.

In a nutshell, leading a microbiome-aware ecotoxicology research (Duperron et al. 2020) is critical, and requires interdisciplinarity, in particular by using the help of microbial ecotoxicology (Hellal et al. 2023), which has collected years of research and experience on the effects of chemicals on microbial communities. This is particularly true in insects, where these studies are still scarce (Antonelli et al. 2022).

### Bridging the gap between understanding chemicals’ mechanisms of actions and identifying their effects on arthropods’ populations

To really understand chemicals’ mechanisms of action in arthropods and the consequences on populations would require to deal with methodological changes and challenges. One major challenge is to causally link the observed effects at the organismal or population levels, with the exposure to a chemical (Soin and Smagghe 2007), and similarly, to link laboratory experimental results with field observations (Kohler et al. 2018; Peterson et al. 2017; Pyle and Ford 2017). A solution to this is to integrate results obtained at different biological levels, from molecular, physiological and organismal levels, to population, species and ecosystem levels (Anton et al. 2011; Peterson et al. 2017). In a review discussing the effects of pesticides on non-target species (including insects), Köhler and Triebskorn (2013) identified two major approaches to bridge this gap: (1) a multi-tiered approach comprising both laboratory experiments, semi-field and field studies, and (2) computational methods aiming at modelling the effects of chemicals at the population level (Köhler and Triebskorn 2013).

Increasing the use of omics data, bioinformatics and modelling in ecotoxicology could help integrate results obtained at different biological levels to bridge the gap between identified chemicals’ mechanisms of actions and their possible effects at higher levels. In this review, only few articles use omic tools (i.e. transcriptomics, metabolomics and lipidomics) to investigate the effects of DEHP or BPA on arthropods (i.e*.* gene expression, metabolite profiles and lipid composition respectively). Several omics techniques can be used depending on the research question. The most used one is probably transcriptomics, which enables for the exploration of differentially expressed genes after exposure to several compounds and/or concentrations. For example, Branco and Lemos found that 48-h exposure to BPA altered the expression of 237 genes, with most of the 96 downregulated genes highly expressed In the midgut, while the expression of only 192 genes was observed after chronic exposure, with upregulated genes mostly composed of ribosome associated genes. In the same study, only 96 genes were differentially expressed after acute exposure (48 h) to DEHP (Branco and Lemos 2014). Metagenomics, in particular amplicon sequencing, can be used to investigate the effects of chemicals and other stresses on microbiota composition. For example, exposure to microplastics can alter midgut microbiota composition in the mosquito species *Aedes aegypti* and *Aedes albopictus* (Edwards et al. 2023). As discussed previously in this review, the concept of Adverse Outcome Pathway can also be used to link the effects of chemicals on molecular actors with adverse outcomes at the organismal and population levels, through Key Events at the physiological level (Ankley et al. 2010). For example, one of the three existing AOP in arthropods makes the link between ecdysone receptor agonism with lethal moulting disruption (Groh et al. 2015). Crane et al. (2022) argued that increasing the use of omics, like transcriptomics, in arthropods ecotoxicology, would be very helpful for identifying chemicals’ molecular initiating event (MIE), a crucial step for understanding their mechanisms of action.

The advent of ecotoxicogenomics opens the way to a deeper understanding of how EDCs and other chemicals could affect invertebrates’ biological functions (Mathiron et al. 2019; Norman and Hughes 2016; Tibbetts and Izzo 2009). Beyond understanding the effects of one chemical in one organism, omic tools coupled to modelling make it possible to extrapolate knowledge of chemicals’ adverse effects on several organisms. For example, Su et al. (2023) used a two compartmental toxicokinetic models to predict the sensitivity of several invertebrate species to a chemical, and Lavado et al. (2022) used Quantitative Structure Activity Relationship (QSAR) models to predict the reproductive toxicity of several organic compounds on the collembola *Folsomia candida*.

In addition, semi-field and field experiments should also be conducted more often in order to better assess the effects of plastic chemicals on arthropods’ populations (Herrero et al. 2014). Indeed, there are very few studies focussing on the effects of plastic chemicals on arthropod populations. To our knowledge, only one study directly investigated the effects of BPA on insects’ populations. Prud’homme et al. (2017) studied the effects of BPA on *Aedes aegypti* life history traits (i.e. wing size, development time, mortality and sex ratio) for six generations, and only found an increased wing size in males from the first generation, suggesting an increased male size, but found no long-term effects on the other investigated traits. At present, the chemicals for which most knowledge has been gathered regarding possible effects on insects’ populations are insecticides. Insect growth regulators (IGRs), designed to target insects’ juvenile hormone and ecdysteroid pathways, have been shown to affect pest insects (Prud’homme et al. 2017), but also to have deleterious effects on non-target species such as pollinators and natural enemies (Tricoire-Leignel et al. 2012). Desneux et al. (2007) also reviewed the sublethal effects of pesticides on beneficial arthropods, taking as an example the honeybee. Several semi-field and field studies have been conducted on honeybees. For example, neonicotinoids have been widely studied for their effects on bees’ foraging behaviours, which can result in negative effects on bees populations (Desneux et al. 2007). In this context, we argue that further field studies and behavioural studies are necessary to better understand the effects of DEHP and BPA on arthropods at the population level.

## Conclusions

In this article, we reviewed the effects of two plastic compounds, DEHP and BPA, on arthropods. Both of these chemicals are found in arthropods, with DEHP concentrations around the μg/g, and BPA concentrations around the ng/g. Exposure to DEHP and BPA induces general toxicity in arthropods, but also more specific effects like alteration of metabolism, and nervous and endocrine systems, resulting in effects on development, fecundity and behaviour. Despite the increasing number of articles published on DEHP and BPA effects in arthropods in recent years, the mechanisms of action of those chemicals in arthropods, and the resulting effects at the population level are still unknown.

Overall, we conclude that BPA and DEHP could affect arthropods’ population via four major ways:

1. BPA and DEHP can interfere with biological functions that are critical for the organisms’ or populations’ survival. Indeed, plastic chemicals’ exposure during development could affect, for example, germ line and the reproductive organ development, thus possibly affecting sex-ratio and/or the fertility of individuals. Adult exposure can also affect fecundity, lipid and carbohydrate metabolism, the detoxification and antioxydation systems, and even the immune system, possibly affecting the organisms’ ability to respond to other stressors.
2. DEHP and BPA can interfere with arthropods’ behaviours.
3. DEHP and BPA may affect epigenetic mechanisms in arthropods. As epigenetics induce long-term, and possibly inheritable, changes in gene expression, it could be involved in mediating the effects of plastic chemicals at the population level.
4. Although it is not yet studied, DEHP and BPA may interact with arthropods’ microbiome, which can play critical roles for its hosts, including through detoxification.

More generally, we identify several research gaps and areas of investigation in arthropod ecotoxicology: (1) Gathering more knowledge on arthropods’ hormonal system and how chemicals can affect it; (2) building-up research effort on how chemicals can affect arthropods’ behaviours and increasing the number of semi-field and field studies; (3) gaining more insight on how chemicals can affect epigenetic mechanisms in arthropods, as those mechanisms may be involved in arthropods’ plasticity or adaptation to chemical exposure; (4) integrating the study of how chemicals affect microbiota to arthropod ecotoxicology; and (5) promoting the use of integrative approach, omic tools and modelling to better understand chemicals’ mechanisms of action in arthropods and the possible consequences at the population level.

## Supplementary Information

Below is the link to the electronic supplementary material.

Supplementary file1 (XLSX 42 KB)

Supplementary file2 (XLSX 235 KB)

## Data Availability

All data supporting this review are available within the paper and its Supplementary Information.

## Abbreviations

*20E*: 20 Hydroxyecdysone
*ACC*: Acetyl-CoA carboxylase
*AchE*: Acetylcholine esterase
*ADH*: Alcohol deshydrogenase
*CAT*: Catalase
*CHH*: Crustacean hyperglycaemic hormone
*CHT*: Chitinase
*CYP*: Cytochrome P450
*e74, e75*: Ecdysteroid response genes
*EcR*: Ecdysteroid receptor
*ERR*: Estrogen-related receptor
*GAPDH*: Glyceraldehyde-3-phosphate deshydrogenase
*gat-2*: Gamma-aminobutyric acid transporter subtype 2
*GIH*: Gonad-inhibiting hormone
*GS*: Glutamine synthetase
*GST*: Glutathione S transferase
*HR3*: An orphan nuclear receptor
*HSC*: Heat shock protein, constitutively expressed isoform
*HSP*: Heat shock protein
*ILP*: Insulin lile peptide
*IR*: Insulin receptor
*JH*: Juvenile hormone
*MDA*: Malondialdehyde
*MIH*: Moult-inhibiting hormone
*PK*: Pyruvate kinase
*PKF*: 6-Phosphofructokinase 1
*PO*: Phenoloxidase
*SOD*: Superoxide dismutase
*SP*: Serine type endopeptidase
*T-AOC*: Total anti-oxidant capacity
*USP*: Ultraspiracle
*VTG*: Vitellogenin
